# Epithelial zinc finger protein in lung adenocarcinoma: prognostic biomarker with molecular and clinical implications

**DOI:** 10.1186/s41065-025-00476-7

**Published:** 2025-06-18

**Authors:** Xiaofen Wen, Jianling Zhu, Didi Xi, Minna Chen, De Zeng, Wenwu Xue, Danxia Lin, Jiaxin Shen

**Affiliations:** 1 Department of Medical Oncology, Cancer Hospital of Shantou University Medical College, Shantou, Guangdong 515031 China; 2 Department of Pathology, Cancer Hospital of Shantou University Medical College, Shantou , 515031 Guangdong China; 3 Department of Oncology and Hematology, Dongguan Binhai Bay Central Hospital, Dongguan , Guangdong 523903 China; 4 Department of Hematology, The First Affiliated Hospital of Shantou University Medical College, Shantou, Guangdong 515031 China

**Keywords:** EZF/KLF4, Prognostic Marker, Lung Adenocarcinoma, Tumor Immunity, Nomogram

## Abstract

**Objective:**

This study aimed to evaluate the prognostic significance of epithelial zinc finger protein (EZF/KLF4) in lung adenocarcinoma (LAC) and explore its potential roles in tumor progression and immune regulation.

**Methods and Materials:**

EZF expression and its associations with clinical characteristics were analyzed using TCGA and GEO datasets, and validated by immunohistochemistry in 25 paired LAC and adjacent normal tissues. Mechanistic insights were investigated through protein–protein interaction networks, gene set enrichment analysis (GSEA), DNA methylation profiling, and immune cell infiltration analysis via single-sample GSEA. A prognostic nomogram incorporating EZF expression, pT and pN stages, and residual tumor status was constructed using Cox regression modeling.

**Results:**

EZF expression was significantly downregulated in LAC tissues compared to normal tissues across multiple cohorts (*P* < 0.001), yet paradoxically associated with advanced tumor stages and worse overall, disease-specific, and progression-free survival. Functional analyses revealed EZF-associated pathways enriched in immune modulation. EZF expression correlated strongly with infiltrating immune cells, including NK cells, eosinophils, mast cells, and neutrophils. Hypomethylation of the EZF promoter was linked to poor prognosis. The constructed nomogram exhibited strong predictive accuracy for patient outcomes.

**Conclusion:**

EZF functions as a context-dependent regulator in LAC and may act as a prognostic biomarker by modulating tumor-immune interactions. These findings offer novel insights into the integration of molecular and immune features for personalized risk stratification and therapeutic guidance in LAC.

**Supplementary Information:**

The online version contains supplementary material available at 10.1186/s41065-025-00476-7.

## Introduction

Lung cancer continues to pose a major global health challenge, with lung adenocarcinoma (LAC) representing the most prevalent subtype of non-small cell lung cancer (NSCLC), accounting for approximately 40% of cases [1, 2]. The asymptomatic nature of early-stage LAC often delays diagnosis, precluding timely surgical intervention. Despite advancements in treatment, including targeted therapies, the overall survival (OS) rates for LAC remain suboptimal. Therapeutic breakthroughs targeting driver mutations, such as BRAF V600E, EGFR, ALK, and MET, have shown promise [3–5]. However, for patients without actionable mutations or access to targeted therapies, traditional chemotherapy and radiotherapy are the primary options, underscoring the need for novel approaches.

In the past decades, immunotherapy has revolutionized the management of LAC by harnessing the body's immune system to fight cancer. Notably, immune checkpoint blockade, a promising form of immunotherapy, targets crucial surface proteins on T cells that inhibit the immune response against malignant antigens [6]. Inhibitors targeting programmed death-1 (PD-1) or programmed death ligand 1 (PD-L1) have demonstrated efficacy as monotherapy or in combination with traditional therapies for LAC [7]. Despite these significant advancements, a considerable proportion of patients exhibit resistance to immunotherapy agents due to various molecular mechanisms [8]. This highlights the critical need for biomarkers to predict prognostic significance and evaluate immunotherapy efficacy.

Epithelial zinc finger protein (EZF, also known as KLF4) is a transcription factor with dual roles in transcriptional activation and repression [9, 10]. In the context of LAC, EZF has demonstrated multifaceted roles, including tumor-suppressive functions through apoptosis induction and proliferation inhibition, alongside tumor-promoting properties via its regulation of oncogenic pathways [11]. However, a comprehensive understanding of how EZF is orchestrated in LAC and its impact on the effectiveness of immunotherapy still requires further investigation. This study seeks to elucidate the role of EZF in LAC, particularly its association with clinical characteristics and immune modulation. By investigating its prognostic value and therapeutic potential, we aim to provide molecular insights that could advance precision medicine strategies in LAC.

## Materials and methods

### Cohort resources

mRNA profiles and clinical parameters of LAC patients and healthy individuals were collected from publicly available sources, including the Gene Expression Omnibus (GEO) database (http://www.ncbi.nlm.nih.gov/geo/), the Genotype-Tissue Expression (GTEx) project (https://xenabrowser.net/datapages/), and the Cancer Genome Atlas (TCGA) database (https://portal.gdc.cancer.gov) (Supplementary Material 1).

RNA-sequencing data for a patient cohort (*n* = 594) and corresponding adjacent normal tissues (*n* = 58) were obtained from the TCGA dataset. To increase the number of normal samples, transcriptomic profiles of normal lung tissues from the GTEx project (*n* = 288) were included. For external validation, two GEO datasets were used: GSE10072, comprising LAC tissues (*n* = 58) and controls (*n* = 48), and GSE115002, including LAC tissues (*n* = 52) and normal controls (*n* = 51). Additionally, two other GEO datasets were used for survival validation: GSE68465 and GSE68571.

For TCGA data, raw count data were normalized and transformed into transcripts per million (TPM) using the following procedure: raw read counts were divided by gene length (in kilobases) to obtain reads per kilobase (RPK); RPK values were then divided by the sum of all RPK values for that sample and multiplied by 10^6 to calculate TPM; finally, TPM values were log2-transformed [log2(TPM + 1)] prior to downstream analysis. Gene annotations were harmonized using the GENCODE v22 reference. All RNA-seq data were processed using a unified pipeline (STAR-aligned BAM files and HTSeq-counts).

For GEO datasets, all data were downloaded in MINiML format, which includes complete GSE records containing platform information, sample metadata, and expression matrices. If raw expression values had not been pre-processed, log2 transformation was performed. For datasets that were not normalized, we applied the quantile normalization method using the normalize.quantiles function from the preprocessCore R package. Probes were mapped to gene symbols using the corresponding platform annotation files. When multiple probes corresponded to a single gene, their expression values were averaged. Probes mapping to multiple genes were excluded.

For pan-cancer analysis, TPM-formatted RNA-seq data were also retrieved from UCSC Xena and GTEx portals.

### Collection of LAC samples and immunohistochemistry analysis

The collection and use of human tissues were proved by the Ethics Committee of Cancer Hospital of Shantou University (Approval Number: 2021042) and was performed in strict adherence to the Declaration of Helsinki. From January 2020 to December 2022, a total of 25 paired samples were obtained from the Pathology Department, Cancer Hospital of Shantou University, consisting of paraffin-preserved LAC tissues with their corresponding adjacent para-cancerous tissues. Basic information of the patients such as gender and age is collected (Supplementary Material 2). Hematoxylin and eosin staining were conducted on each sample, and reviewed by two pathologists, to confirm the diagnosis. Micro-dissection techniques were employed to ensure a tumor cell content of at least 70% in the samples, while the control group was histologically confirmed across non-cancerous samples.

LAC and adjacent tissues were embedded in paraffin, followed by sectioning into slides with a thickness of 5 μm. Subsequently, the slides were deparaffinized incubated with a 3% H2O2 solution for 10 min to block endogenous peroxidase activity. After antigen retrieval, the slides were incubated with the anti-EZF rabbit monoclonal antibody (#ab215036, 1:200 dilution, abcam, Cambridge, UK) overnight at 4 °C, followed by incubation with a secondary antibody (1: 1000 dilution, Beyotime, Shanghai, China) for 20 min at room temperature. After color development using a diaminobenzidine solution, the slides were washed with purified water and counterstained with hematoxylin, and finally observed under a microscope.

### Evaluation of functional enrichment

TCGA LAC cohort was categorized based on the median EZF expression score. Differentially expressed genes (DEGs) were analyzed using R package DESeq2 [12, 13], with a threshold of statistical significance at an adjusted *P* < 0.05 and an absolute log2 fold change (FC) > 1. Spearman's correlation analysis was conducted to investigate the association between 10 leading DEGs and EZF expression levels. Subsequently, these correlated genes were employed to build a protein–protein interaction (PPI) network by extracting relevant data from the Retrieval of Interacting Genes (STRING) online database, with a threshold of confidence score > 0.7 and default variables applied. The network was visualized and further analyzed to screen the hub genes in Cytoscape software [14]. The"CytoHubba"was plugged in to achieve the top 10 hub genes among DEGs [15].

Furthermore, functional enrichment of EZF was performed using the R packages"ggplot2″ and"clusterProfiler [16, 17]”. These analyses encompassed gene set enrichment analysis (GSEA), Kyoto Encyclopedia of Genes and Genomes (KEGG) analysis, and Gene Ontology (GO). Adjusted P values were calculated using the False Discovery Rate (FDR) estimation with a threshold set at FDR < 0.25. A significance threshold of less than 0.05 for the adjusted P value was adopted.

### Analysis of immune cell infiltration level

Single-sample GSEA (ssGSEA) technique was employed to assess the immune infiltration extent in LAC tissues among 24 immune cell types through the GSVA R package [18, 19]. Spearman's correlation was used to investigate the underlying relation of immune cell abundance with EZF expression. The Wilcoxon rank-sum test was employed to evaluate variances in immune infiltration levels between groups with different levels of EZF expression levels. To predict how EZF involved or correlated with immune infiltration levels, Tumor Immune Estimation Resource 2.0 (TIMER2.0; http://timer.cistrome.org/) was used [20, 21]. Tumor Mutational Burden (TMB) enrichment score was introduced to reflect tumor immunity and assessed specific correlation with EZF expression using the R package"ggstatsplot". Additionally, immune modulator analysis was performed using the Tumor Immune System Interacting Database (TISIDB), which incorporated data sourced from TCGA [22].

### Analysis of DNA methylation

UALCAN database was applied to scrutinize the methylation of EZF promoter [23, 24]. Additionally, the prognostic significance of EZF methylation levels was assessed using MethSurv database [25].

### Survival analysis

The log-rank test was performed using the classical Kaplan–Meier (KM) method to assess clinical outcomes of the patients, with the median EZF expression as the cutoff value. Univariate and multivariate Cox regression analyses were conducted to investigate the influence on patient outcomes. To identify independent prognostic factors, significant features identified in univariate Cox regression analysis (*P* < 0.1) underwent multivariate Cox regression analysis. The results were visually presented in a forest plot using ggplot2.

### Construction and validation of nomogram

A nomogram was constructed using R package RMS to identify the quantitative contribution of independent prognostic factors from multivariate Cox analysis and to determine the probabilities of 1-, 3-, and 5-year OS. The concordance index (C-index) was calculated, and calibration plots were drawn to evaluate the performance and discriminatory ability of the model. Time-dependent receiver-operating characteristic (ROC) curves were plotted to evaluate the predictive accuracy of the nomogram model.

### Statistical analysis

R software (version 4.3.1) was employed to conduct the statistical analyses in this study. Two sided tests were conducted, and statistical significance was attributed to *P* value < 0.05. The significances of EZF expression in paired and unpaired tissues were assessed using paired sample t-test and Wilcoxon rank-sum test, respectively. Wilcoxon rank-sum test and logistic regression were performed to determine the association between EZF expression and clinical features.

## Results

### Analysis of EZF Expression in pan-cancer

**Fig. 1:**
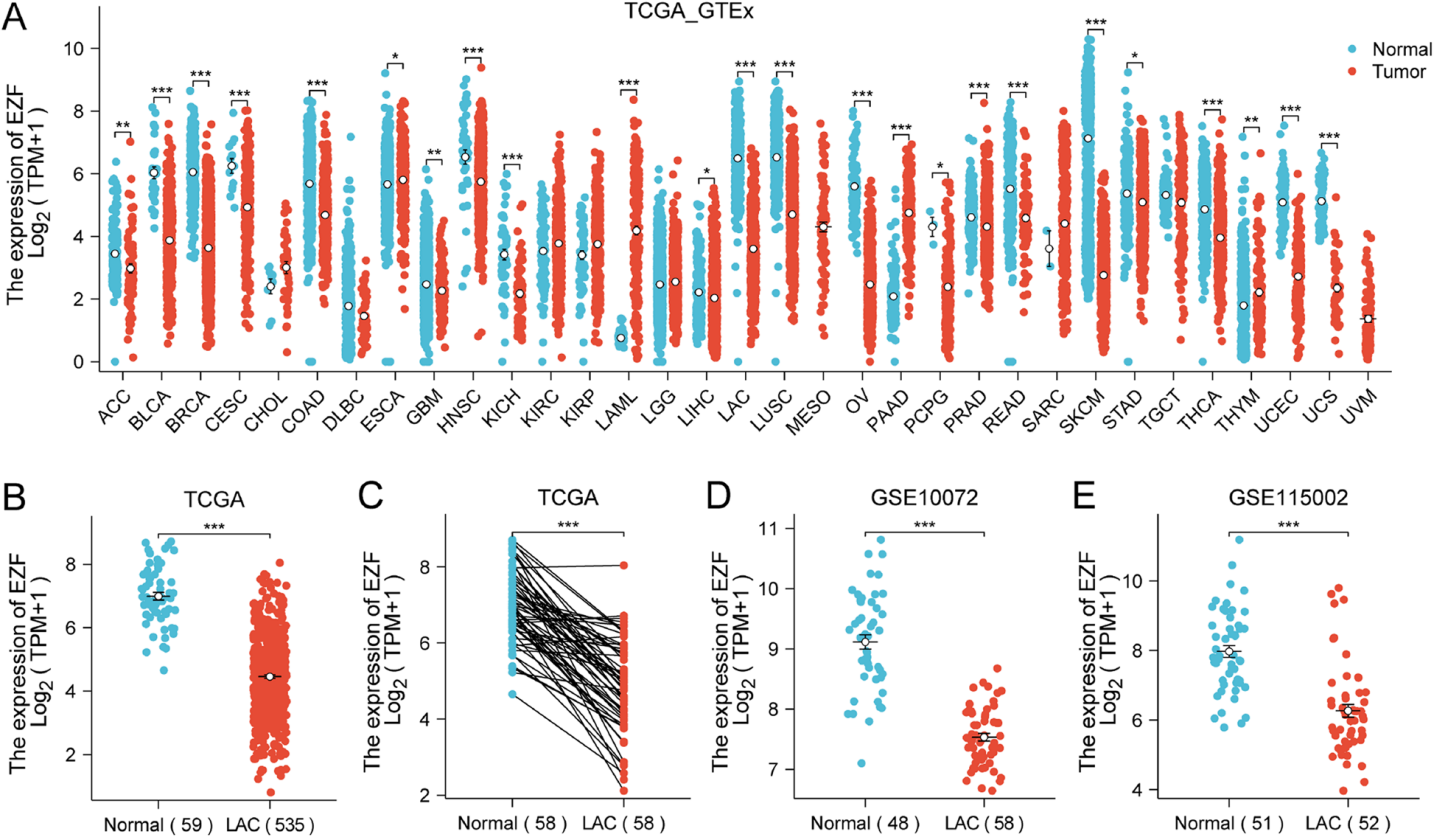
The expression level of EZF in pan-cancer including LAC. Expression of EZF in different types of tumors compared with normal tissues in TCGA and GTEx databases (**A**); in non-matched (**B**) and matched (**C**) normal lung tissues vs LAC from TCGA; in normal vs LAC tissues from datasets GSE10072 (**D**), and GSE115002 (**E**). TCGA, The Cancer Genome Atlas; GTEx, Genotype Tissue Expression Project; GEO, Gene Expression Omnibus. *P < 0.05, **P < 0.01, ***P < 0.001

In the context of pan-cancer analysis, EZF expression exhibited a general downregulation across 15 different cancer types (Fig. 1A). Notably, the expression of EZF in LAC was significantly lower compared to non-cancerous lung tissues (Fig. 1B). This observation was further validated in a cohort of 58 paired LAC tissues, where EZF expression exhibited a notable reduction (Fig. 1C). Consistently, the EZF expression was markedly lower in LAC compared to the control groups in GSE10072 (Fig. 1D) and GSE115002 datasets (Fig. 1E).

**Fig. 2:**
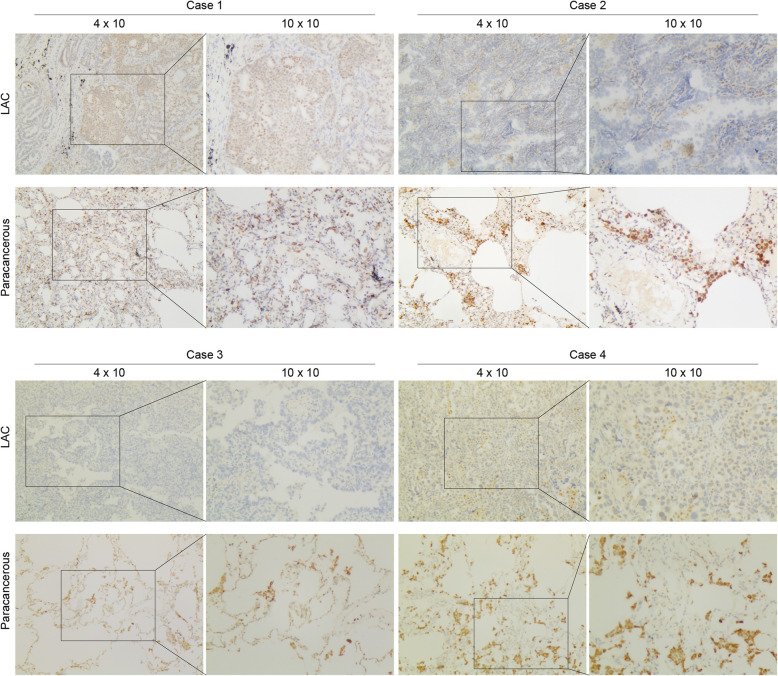
Representative images of EZF expression in LAC tissues and matched paracancerous tissues. Original magnifications 40 × and 100 × (inset panels)

To examine the expression of EZF, we performed immunohistochemical (IHC) staining on a cohort consisting of 25 cases of LAC and their corresponding non-cancerous samples. Two samples were excluded due to the absence of cancerous tissues in the paraffin-embedded specimens. Ultimately, a total of 23 patients were included. Consistent with the in silico data, EZF exhibited significantly reduced staining density in LAC, suggesting that EZF is lowly expressed in cancerous tissues compared to normal lung tissue (Fig. 2). To further validate these findings, additional evidence from the Human Protein Atlas (HPA) database has been incorporated, demonstrating similar expression patterns of EZF in both normal lung tissue and LAC, as shown in Supplementary Figure S1.

### Relationship between EZF expression and clinicopathological characteristics of LAC

**Fig. 3:**
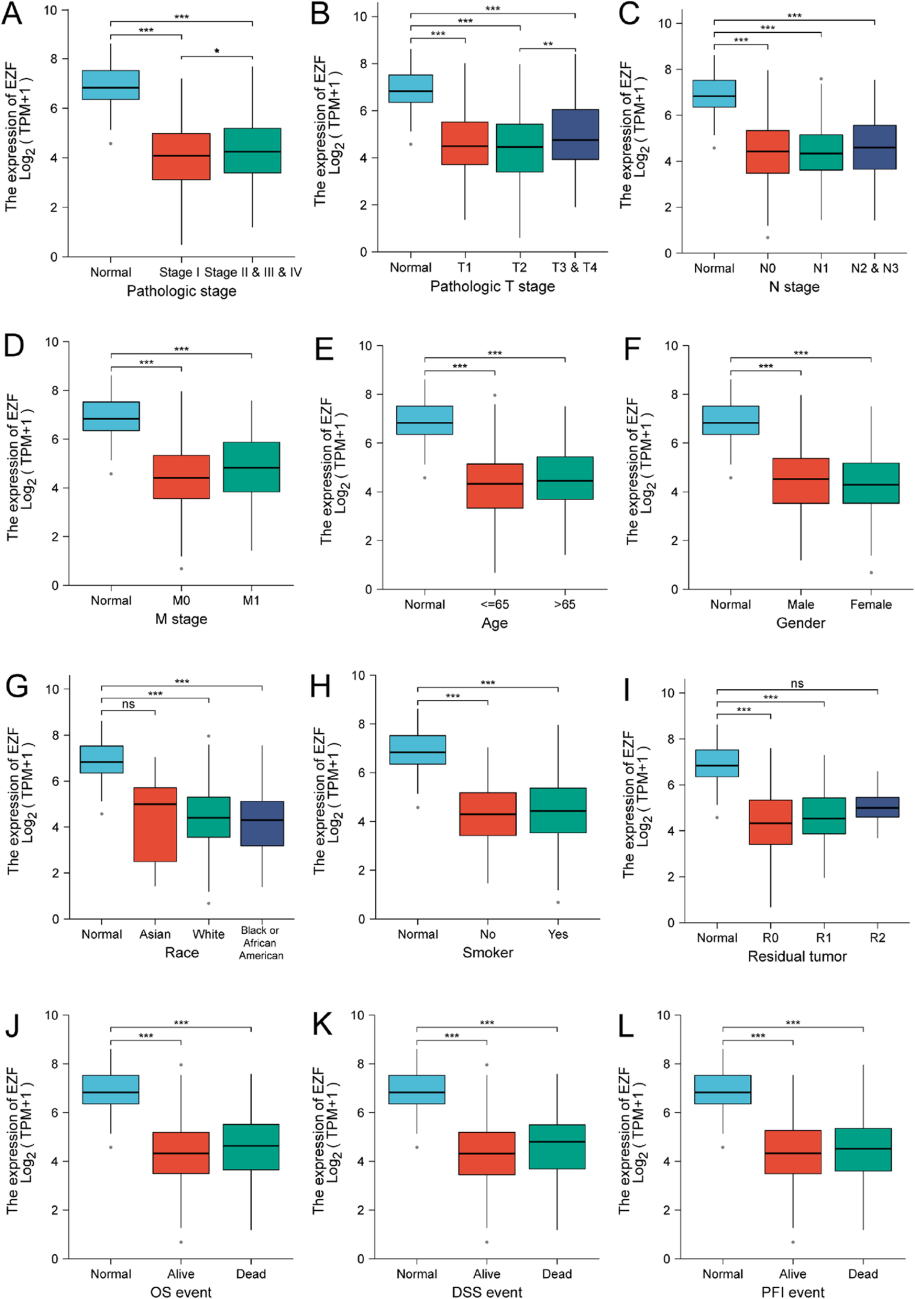
Associations between EZF expression and clinicopathological characteristics. Data are shown for (**A**) pathologic stage; (**B**) T stage; (**C**) N stage; (**D**) M stage; (**E**) age; (**F**) gender; (**G**) race; (**H**) smoker; (**I**) residual tumor; (**J**) OS event; (**K**) DSS event; and (**L**) PFI event. OS, overall survival; DSS, disease-specific survival; PFI, progression-free interval. *P < 0.05, **P < 0.01, and ***P < 0.001

Next, TCGA database was used to investigate the impact of different pathologic features on EZF transcription in LAC samples. EZF expression consistently exhibited reduced levels in LAC patients compared to controls across different sub-groups (Table 1, Fig. 3). Sub-group analyses, encompassing pathologic stage, TNM classification, age, gender, ethnicity, smoking history, residual tumor, and survival factors, consistently demonstrated a significant decrease in EZF expression among LAC patients compared to the normal control group. Furthermore, univariate logistic regression analysis revealed statistically significant differences between groups characterized by distinct EZF expression levels for gender (male vs. female) and T stage (T3,4 vs. T1,2), with Odds Ratios (ORs) of 1.538 (95% CI = 1.094—2.167) and 1.875 (95% CI = 1.117—3.209), respectively (Table 2).

**Table 1:** Clinicopathological characteristics of high- and low-EZF expression groups

Characteristics	Low expression of EZF	High expression of EZF	p
n	267	268	
Age, median (IQR)	65 (59, 71)	67 (59, 73)	0.183
Age, n (%)			0.376
< = 65	135 (26.2%)	120 (23.3%)	
> 65	127 (24.6%)	134 (26%)	
T stage, n (%)			0.041
T1	91 (17.1%)	84 (15.8%)	
T2	151 (28.4%)	138 (25.9%)	
T3	15 (2.8%)	34 (6.4%)	
T4	10 (1.9%)	9 (1.7%)	
N stage, n (%)			0.508
N0	171 (32.9%)	177 (34.1%)	
N1	50 (9.6%)	45 (8.7%)	
N2	34 (6.6%)	40 (7.7%)	
N3	2 (0.4%)	0 (0%)	
M stage, n (%)			0.257
M0	180 (46.6%)	181 (46.9%)	
M1	9 (2.3%)	16 (4.1%)	
Pathologic stage, n (%)			0.234
Stage I	156 (29.6%)	138 (26.2%)	
Stage II	57 (10.8%)	66 (12.5%)	
Stage III	41 (7.8%)	43 (8.2%)	
Stage IV	9 (1.7%)	17 (3.2%)	
Primary therapy outcome, n (%)			0.13
PD	30 (6.7%)	41 (9.2%)	
SD	22 (4.9%)	15 (3.4%)	
PR	1 (0.2%)	5 (1.1%)	
CR	169 (37.9%)	163 (36.5%)	
Gender, n (%)			0.017
Female	157 (29.3%)	129 (24.1%)	
Male	110 (20.6%)	139 (26%)	
Race, n (%)			0.762
Asian	3 (0.6%)	4 (0.9%)	
Black or African American	30 (6.4%)	25 (5.3%)	
White	203 (43.4%)	203 (43.4%)	
Residual tumor, n (%)			0.386
R0	183 (49.2%)	172 (46.2%)	
R1	5 (1.3%)	8 (2.2%)	
R2	1 (0.3%)	3 (0.8%)	
Histological type, n (%)			0.623
L-ACA	9 (1.8%)	9 (1.8%)	
L-ADM	58 (11.8%)	51 (10.4%)	
L-ADC-NOS	170 (34.6%)	170 (34.6%)	
IMA	1 (0.2%)	4 (0.8%)	
AIS or MIA	11 (2.2%)	8 (1.6%)	
Anatomic neoplasm subdivision, n (%)			0.473
Left	98 (18.8%)	107 (20.6%)	
Right	162 (31.2%)	153 (29.4%)	
Anatomic neoplasm subdivision2, n (%)			0.53
Central Lung	27 (14.3%)	35 (18.5%)	
Peripheral Lung	63 (33.3%)	64 (33.9%)	
number_pack_years_smoked, n (%)			0.28
< 40	85 (23%)	103 (27.9%)	
> = 40	93 (25.2%)	88 (23.8%)	
Smoker, n (%)			0.422
No	41 (7.9%)	34 (6.5%)	
Yes	218 (41.8%)	228 (43.8%)	
OS event, n (%)			0.063
Alive	182 (34%)	161 (30.1%)	
Dead	85 (15.9%)	107 (20%)	
DSS event, n (%)			0.023
Alive	202 (40.5%)	177 (35.5%)	
Dead	49 (9.8%)	71 (14.2%)	
PFI event, n (%)			0.271
Alive	161 (30.1%)	148 (27.7%)	
Dead	106 (19.8%)	120 (22.4%)	

*IQR* Interquartile range, *NOS* Not otherwise specified, *L-ACA* Lung Acinar Adenocarcinoma, L*-ADM* Lung Adenocarcinoma Mixed Subtype, *L-ADC-NOS* Lung Adenocarcinoma-Not Otherwise Specified, *IMA* Invasive Mucinous Adenocarcinoma, *AIS* Adenocarcinoma in Situ, *MIA* Minimally Invasive Adenocarcinoma

**Table 2:** Associations of EZF expression with clinical features in LAC patients (*n* = 535)

Characteristics	Total (N)	Odds Ratio (OR)	P value
T stage (T3&T4 vs. T1&T2)	532	1.875 (1.117–3.209)	0.019
N stage (N2&N3 vs. N0&N1)	519	1.106 (0.679–1.806)	0.685
M stage (M1 vs. M0)	386	1.768 (0.776–4.275)	0.185
Pathologic stage (Stage III&Stage IV vs. Stage I&Stage II)	527	1.253 (0.823–1.915)	0.294
Primary therapy outcome (SD&PR&CR vs. PD)	446	0.697 (0.415–1.161)	0.168
Gender (Male vs. Female)	535	1.538 (1.094–2.167)	0.014
Race (Asian&Black or African American vs. White)	468	0.879 (0.512–1.501)	0.636
Age (> 65 vs. < = 65)	516	1.187 (0.840–1.678)	0.331
Residual tumor (R1&R2 vs. R0)	372	1.951 (0.726–5.768)	0.198
Anatomic neoplasm subdivision (Right vs. Left)	520	0.865 (0.608–1.230)	0.419
Anatomic neoplasm subdivision2 (Peripheral Lung vs. Central Lung)	189	0.784 (0.423–1.441)	0.434
number_pack_years_smoked (> = 40 vs. < 40)	369	0.781 (0.518–1.175)	0.236
Smoker (Yes vs. No)	521	1.261 (0.773–2.070)	0.354

### Analysis of the PPI network of the DEGs in LAC

**Fig. 4:**
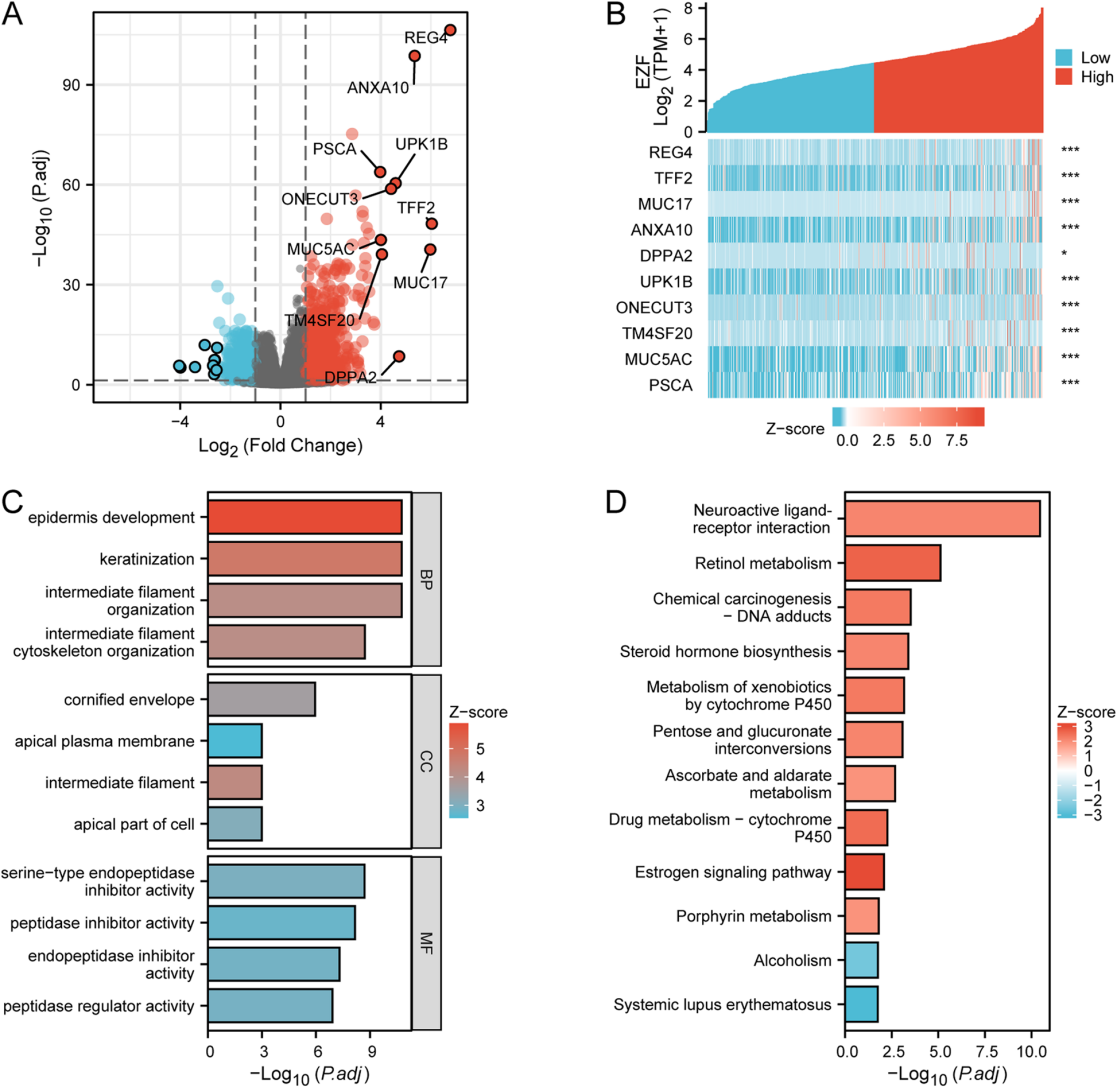
EZF-related DEGs and functional enrichment analysis of EZF in LAC using GO and KEGG enrichment analysis. **A** Volcano plot of DEGs. Blue and red dots indicate the significantly down-regulated and up-regulated DEGs, respectively. **B** Heatmap of correlation between EZF expression and the top 10 DEGs. **C** GO analysis of DEGs. **D** KEGG analysis of DEGs. GO, Gene Ontology; KEGG, Kyoto Encyclopedia of Genes and Genomes; DEGs, differentially expressed genes

By conducting a comparative analysis of gene expression profiles among LAC exhibiting diverse levels of EZF, we identified 1582 DEGs consisting of upregulated genes (*n* = 1100, 69.53%) and downregulated genes (*n* = 482, 30.47%), meeting the criteria of adjusted *P* values < 0.05 with absolute Log2 FC > 1 (Fig. 4A, Supplementary Material 3). The relationships between EZF and the top ten DEGs are presented (Fig. 4B). To gain a more comprehensive understanding of potential interactions among the top 50 DEGs, we constructed a PPI network and identified a collection of hub genes from this network using STRING. The resulting network displayed a notable level of intricacy, with the identification of the top 10 hub genes (Supplementary Material 4).

### Functional enrichment analyses

**Fig. 5:**
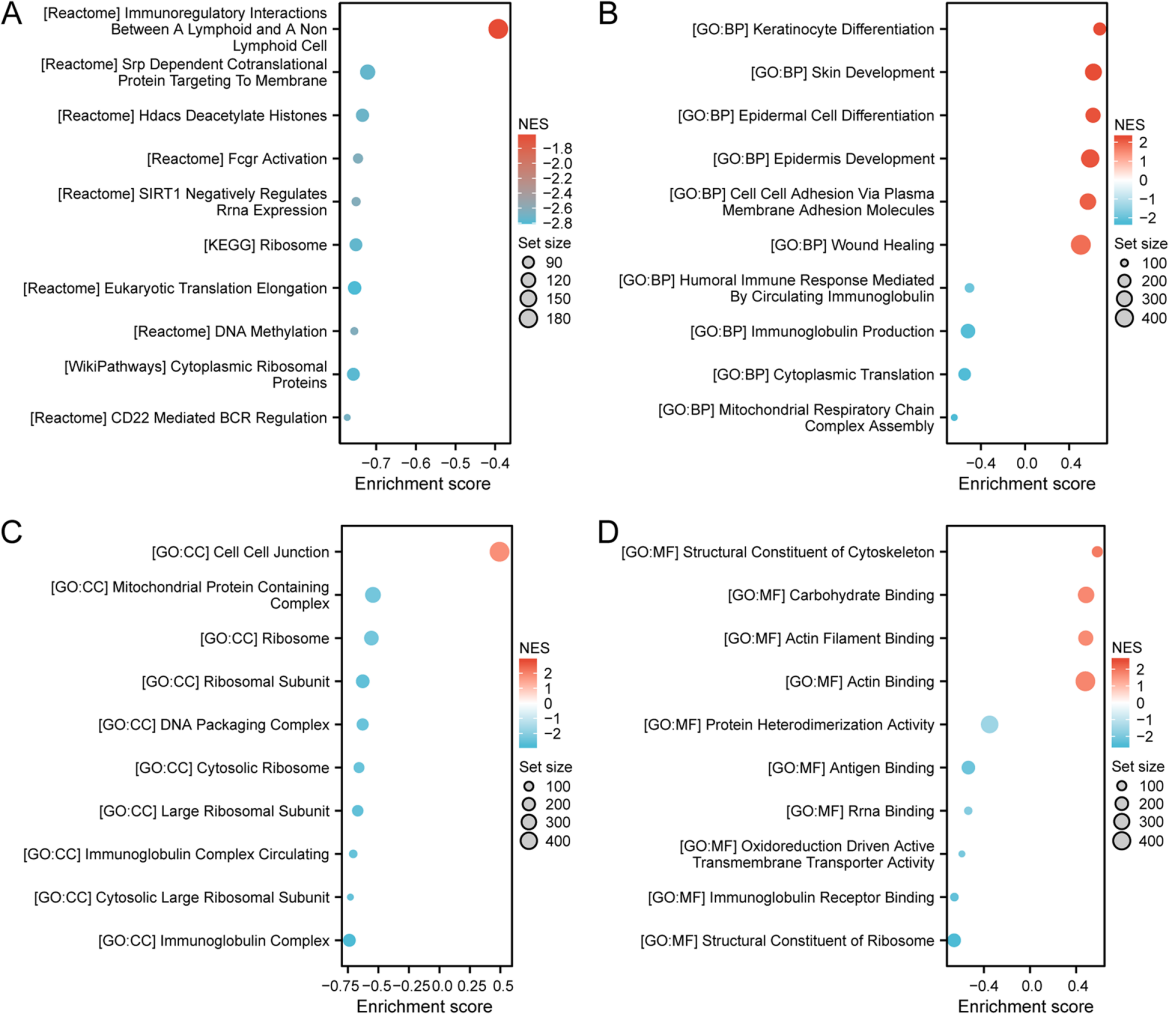
GSEA analysis for DEGs. GSEA analysis of all canonical pathways gene sets (**A**), bp (**B**), cc (**C**), and mf (**D**) of GO genes deposited or downloaded from MSigDB. GSEA, gene set enrichment analysis; DEGs, differentially expressed genes; bp, biological process; mf for molecular function; cc for cellular component; GO, Gene Ontology; MSigDB, Molecular Signatures database; NES, normalized enrichment score

To gain a deeper insight into the functional role of identified DEGs, we conducted GO enrichment analysis, including biological processes (BP), cellular components (CC), and molecular functions (MF). This finding revealed significant enrichment in different GO items, including the activity of peptidase inhibitor and epidermis development (Fig. 4C, Supplementary Material 5). The KEGG pathway analysis revealed significant enrichment in neuroactive ligand-receptor interaction and chemical carcinogenesis-related pathways (Fig. 4D, Supplementary Material 5). Moreover, we employed GSEA to predict the functions of the identified DEGs. The results from GSEA indicated substantial enrichment in pathways associated with immunity, including immunoglobulin production and immunoregulatory interactions (Fig. 5, Supplementary Material 6). These findings suggest EZF may modulate the immune microenvironment of LAC tissues.

### Analysis of the impact of DNA methylation level of EZF on the prognosis of LAC patients

**Fig. 6:**
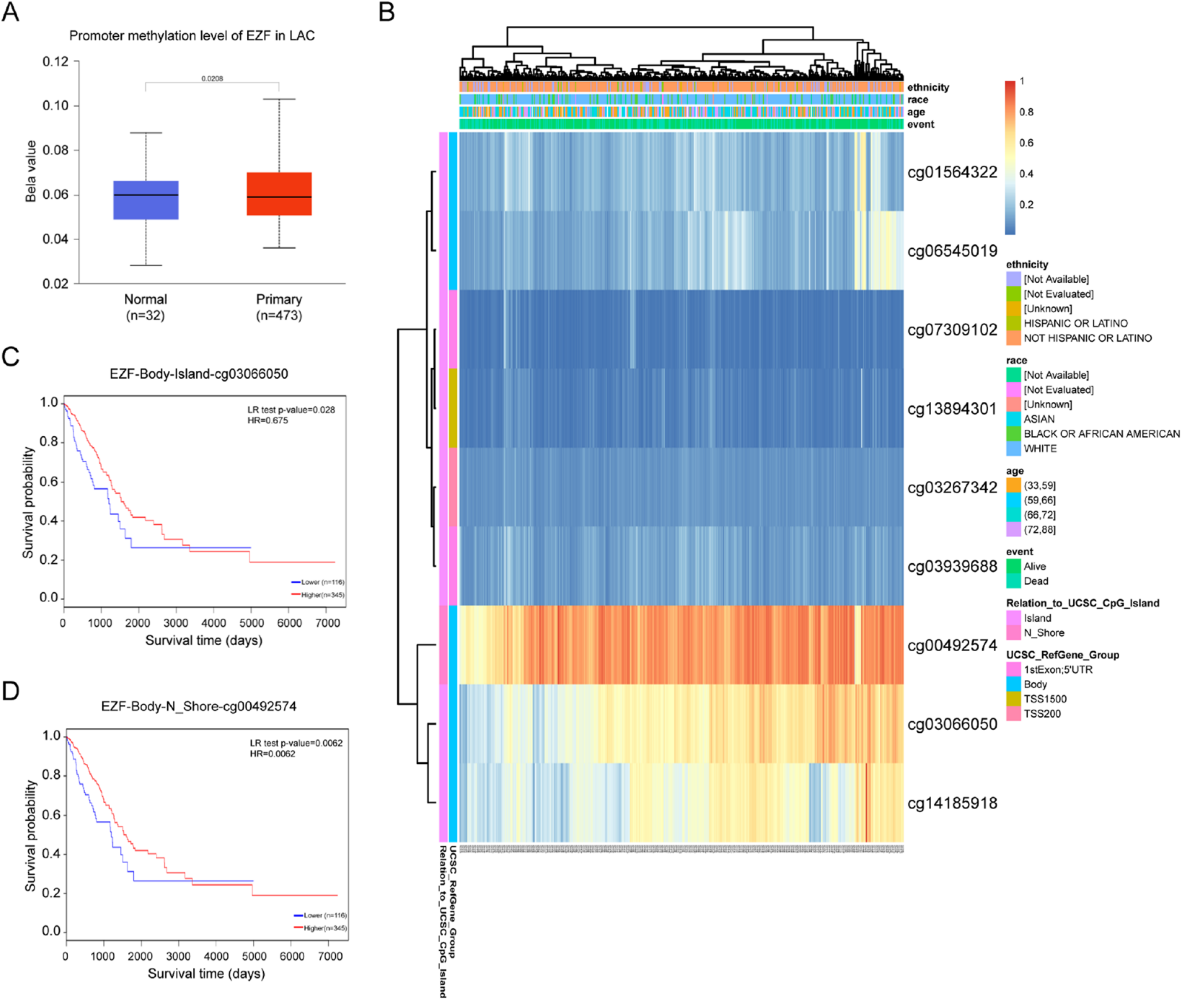
Analysis of the impact of DNA methylation level of EZF on the prognosis of LAC patients. **A** The promoter methylation level of EZF in LAC was obtained from the UALCAN database. **B** Correlation between LAC mRNA expression level and methylation level. **C**, **D** Kaplan–Meier survival curve for methylation sites of EZF

To elucidate the mechanisms contributing to the excessive abundance of EZF in LAC, investigated the relationship between EZF expression and methylation status. Initial findings from the UALCAN database revealed a significantly enhanced level of DNA methylation within the EZF promoter region in LAC tissues (P = 0.0208) (Fig. 6A). Further exploration revealed prevalent hypomethylation across various positions within the DNA sequences of EZF in LAC (Fig. 6B). Notably, the degree of methylation at these positions exhibited a remarkable correlation with patient outcomes. Specifically, patients with reduced EZF methylation demonstrated inferior OS rates compared to those with enhanced levels of EZF methylation (Figs. 6C, D).

## Analysis of correlation between EZF expression and immune infiltration level in LAC

**Fig. 7:**
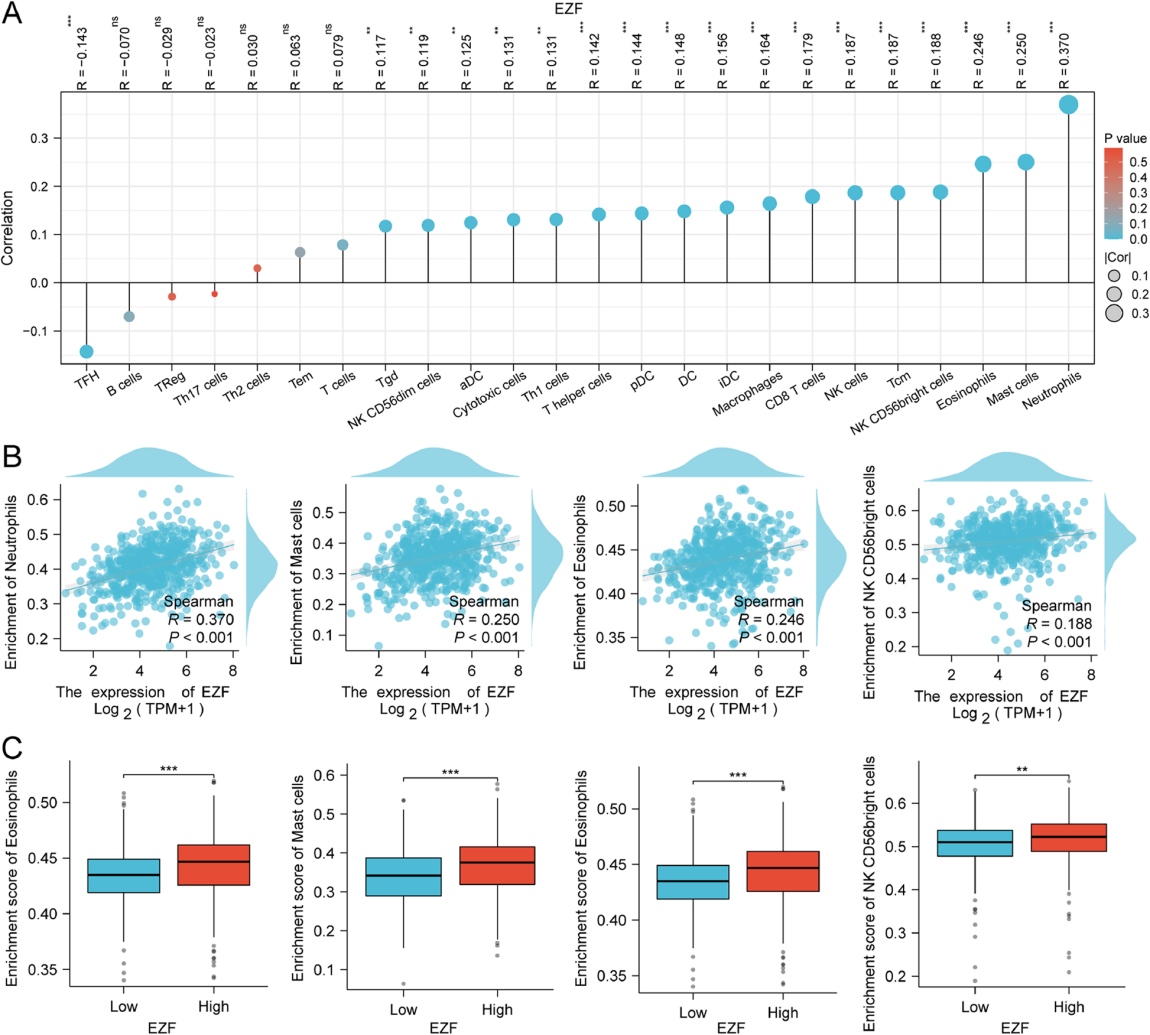
Correlation of EZF expression with immune cell infiltration level in LAC. **A** Correlation between EZF expression and relative abundance of 24 types of immune cell. The size of the dot corresponds to the absolute Spearman’s correlation coefficient values. **B** Correlations between the relative enrichment scores of immune cells (including neutrophils, mast cells, eosinophils, and NK CD56bright cells) and expression of EZF. **C** Comparison of immune infiltration levels of immune cells (including neutrophils, mast cells, eosinophils, and NK CD56bright cells) between high- and low-EZF expression groups. NK cells, are natural killer cells. *P < 0.05, **P < 0.01, and ***P < 0.001

**Fig. 8:**
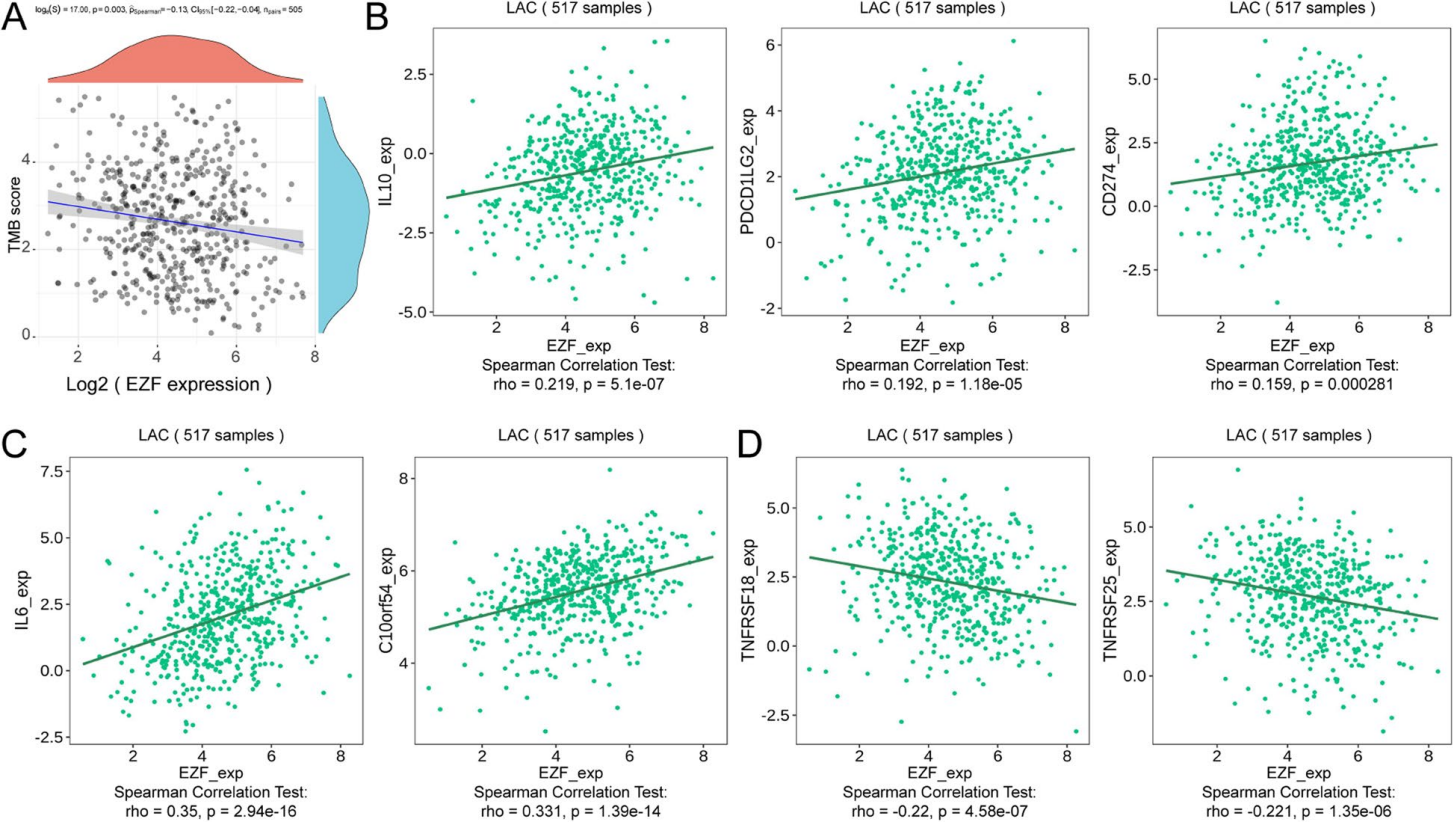
Correlation of EZF expression with immune biomarkers in LAC. **A** Correlations between relative TMB scores and EZF expression. **B** Comparison of immune infiltration levels of 24 types of immune cells between the high- and low-EZF expression groups. **C** Correlations between EZF expression and immunoinhititors including IL10, PDCD1LG2, CD274), and (**D**) immunostimulators including IL6, C10orf54, TNFRSF18 and TNFRSF25

Next, TIMER2.0 was used to unravel the intricate interplay between EZF expression level and infiltrating immune cells in LAC. The results suggested that the expression level of EZF exhibited positive associations with diverse immune cells, including neutrophils, monocytes, CD4 + T cells, macrophages, mast cells, NK cells, and hematopoietic stem cells. Conversely, adverse correlations were observed with Tregs and B cells (Supplementary Material 7). Moreover, significant positive correlations were observed between EZF expression levels and infiltrating immune cells using single-sample GSEA, including neutrophils, eosinophils, NK CD56bright cells, and mast cells (Fig. 7 A, B). The enrichment scores of these cell types were notably higher in the cohort with elevated EZF expression compared to those with low EZF expression (Fig. 7C). Additionally, a negative correlation was observed between TMB scores and EZF expression levels in LAC (r = −0.13, P = 0.003) (Fig. 8A). EZF expression was also positive associations with immune inhibitors such as CD274, PDCD1LG2, and IL10 (Fig. 8B), as well as immune stimulators like C10orf54 and IL6. Conversely, negative correlations were found between EZF expression and TNFRSF25 and TNFRSF18 (Fig. 8C). However, our analyses using both Immune Phenotype Scores (IPS) and Tumor Immune Dysfunction and Exclusion (TiDE) failed to demonstrate a significant association between EZF expression and immune checkpoint inhibitor efficacy, as presented in Supplementary Figure S2.

## Analysis of prognostic significance of EZF in LAC

**Fig. 9:**
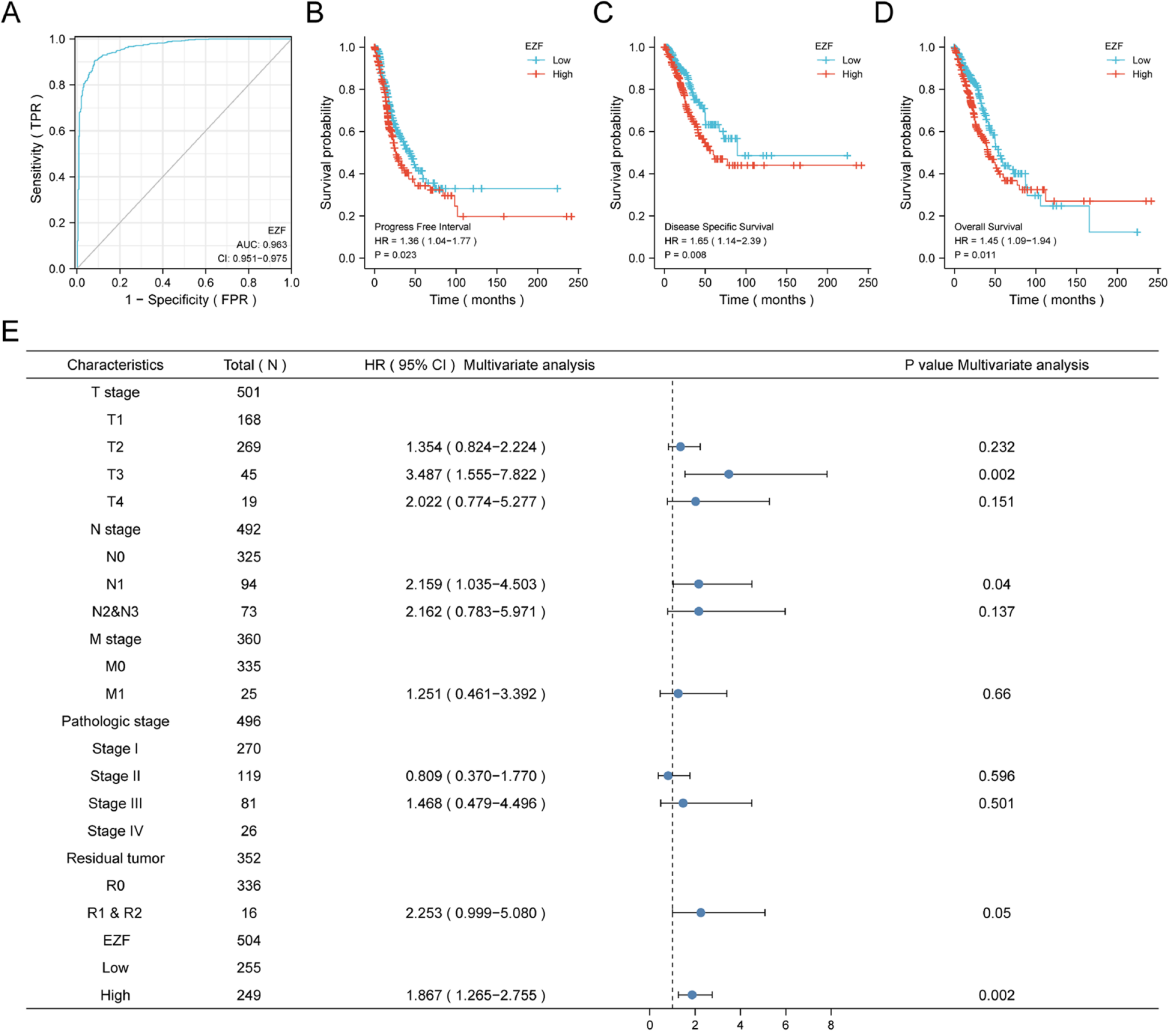
Prognostic values of EZF in patients with LAC. **A** ROC curve to classify LAC vs normal lung tissues in the TCGA database. **B**-**D** Kaplan–Meier analysis of progress-free interval, disease-specific survival, and overall survival in LAC patients with high vs low expression of EZF. **E** Forest map based on multivariate Cox analysis for overall survival. ROC, receiver operating characteristic; HR, hazard ratio; CI, confidence interval

EZF exhibited superior predictive accuracy in distinguishing LAC from normal tissues, as evidenced by the impressive AUC of 0.963 (95% CI = 0.951–0.975) according to ROC (Fig. 9 A). Subsequently, we employed Kaplan–Meier analysis to assess the correlation between EZF expression and clinical outcomes in LAC patients. Stratifying patients into different expression groups using the median as a reference, we observed that those with elevated EZF levels had significantly worse prognosis for the progression-free interval (PFI) (HR = 1.36, 95% CI = 1.04—1.77, P = 0.023), disease-specific survival (DSS) (HR = 1.65, 95% CI = 1.14—2.39, P = 0.008), and OS (hazard ratio [HR] = 1.45, 95% CI = 1.09—1.94, P = 0.011), as illustrated by KM plots when compared with the low expression group (Figs. 9B-D). To further validate the prognostic significance of EZF, we performed external validation using two independent GEO datasets. Consistent with the TCGA findings, high EZF expression was significantly associated with poorer overall survival in both cohorts, reinforcing its potential as a robust and generalizable prognostic biomarker for LAC (Supplementary Figure S3).

**Fig. 10:**
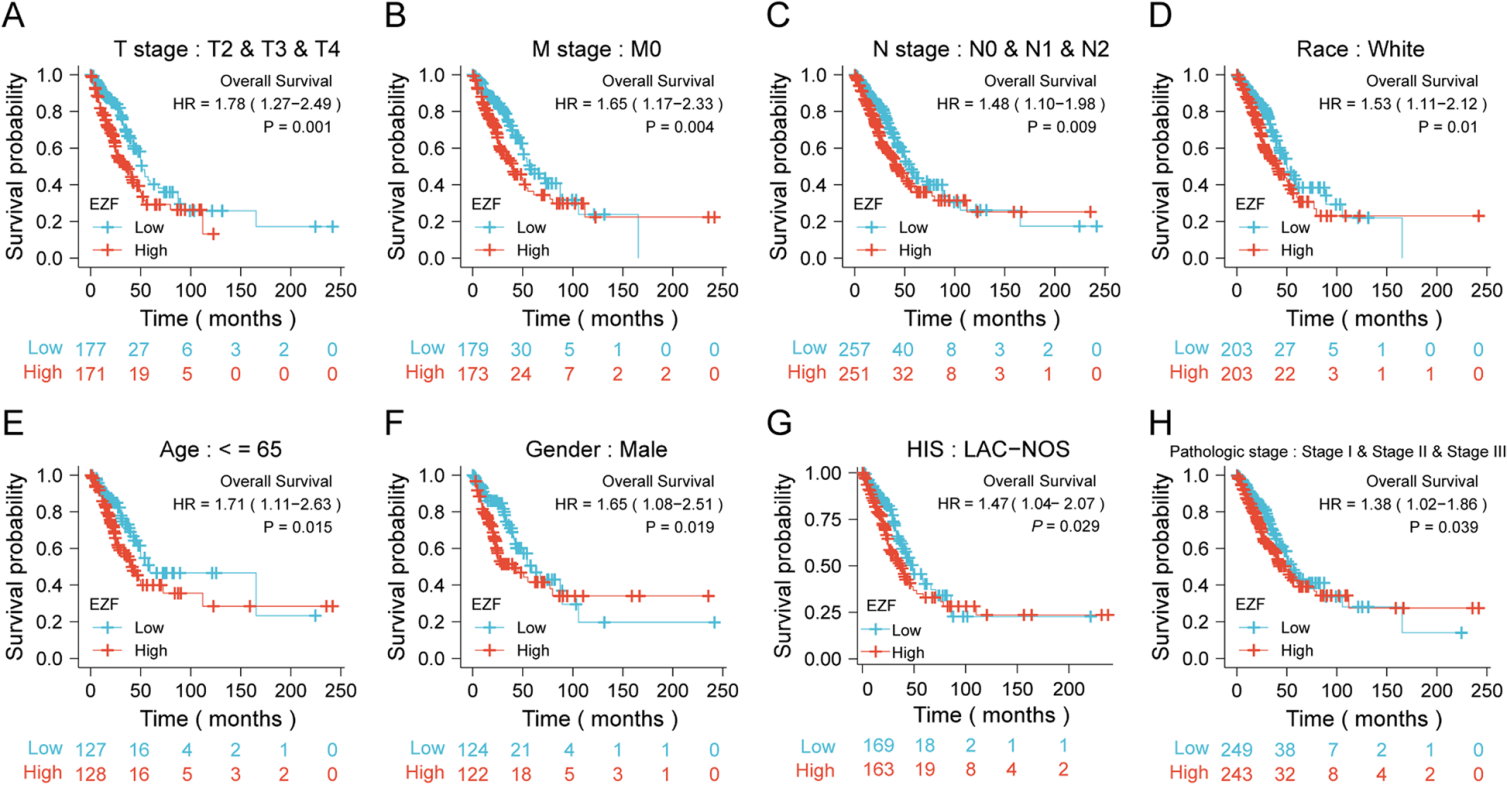
Prognostic values of EZF expression in patients with LAC evaluated by Kaplan–Meier analysis in different clinical subgroups. **A**–**G** OS curves of T2-4, M0, N0-2, white race, age < = 65 years, male, LAC-NOS, and pathologic stage I-III subgroups between high- and low EZF patients with LAC

In the analysis of factors influencing the prognosis of LAC patients, we conducted both univariate and multivariate Cox regression analyses. The multivariate analysis revealed that the N1 stage (adjusted HR = 2.159, 95% CI = 1.035–4.503, P = 0.04) and T3 stage (adjusted HR = 3.487, 95% CI = 1.555–7.822, P = 0.002) were independent risk factors for OS. EZF expression also emerged as an independent prognostic factor with an adjusted HR of 1.867 (95% CI = 1.265–2.755, P = 0.002) (Fig. 9E and Supplementary Material 8). In terms of disease-specific survival, both R1 and R2 tumor status and EZF expression were significant prognostic indicators. Specifically, R1 and R2 tumor status had an adjusted HR of 3.318 (95% CI = 1.115—9.871, P = 0.031), while EZF expression showed an adjusted HR of 1.845 (95% CI = 1.086—3.133, P = 0.024) (Supplementary Material 9). For PFI, three factors were identified as prognostic indicators. R1 and R2 tumor status had an adjusted HR of 3.196 (95% CI = 1.420—7.196, P = 0.005), T3 stage showed an adjusted HR of 2.584 (95% CI = 1.106—6.038, P = 0.028), and EZF expression had an adjusted HR of 1.547 (95% CI = 1.057—2.265, P = 0.025) (Supplementary Material 10). Furthermore, the prognostic significance of EZF was consistently observed in various subgroups, indicating an association between high EZF expression and adverse clinical outcomes. These subgroups included stages of T2/3/4 stage, M0, N0/1/2, white race, age ≤ 60 years, male gender, and pathologic stages I-III (all *P* < 0.05) (Fig. 10, Supplementary Material 11).

## Development and verification of the nomogram for predication of prognostic in LAC patients

**Fig. 11:**
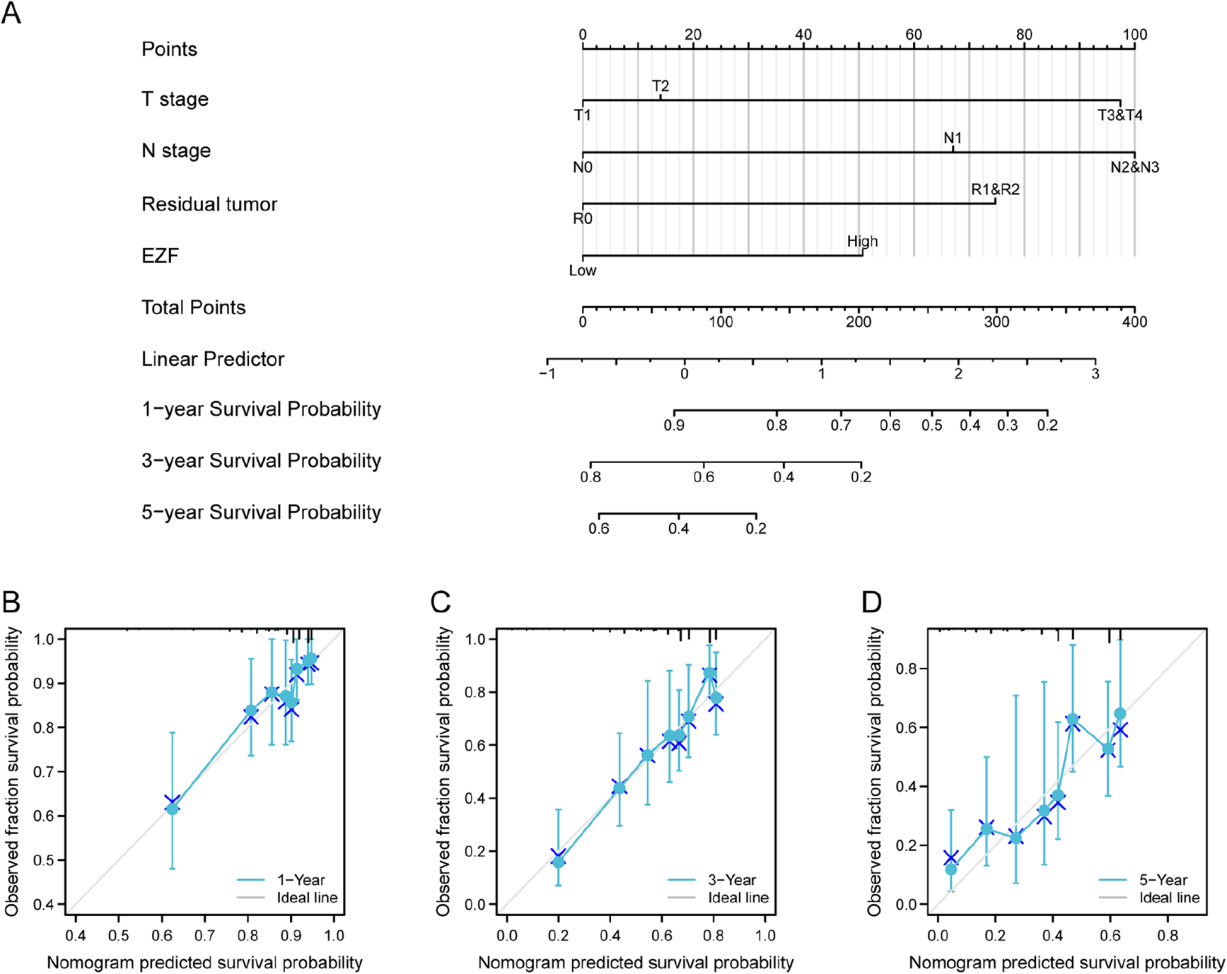
A nomogram and calibration curves were used to predict 1-, 3-, and 5-year OS rates of patients with LAC. **A** A nomogram for prediction of 1-, 3-, and 5-year OS rates of patients with LAC. **B**–**D** Calibration curves of the nomogram's predictions for the 1-, 3-, and 5-year OS rates in patients with LAC

Next, a nomogram incorporating the independent prognostic factors was constructed, for predicting the OS of the patients with LAC. The nomogram represents the contribution of all factors, with a lower total number of points corresponding to a more favorable outcome (Fig. 11A). To assess the predictive performance of the nomogram, calibration curves were generated (Fig. 11B-D). The bootstrap-corrected C-index was 0.692 (95% CI = 0.667—0.716), suggesting a moderate level of predictive performance for OS in LAC. Furthermore, time-dependent receiver operating characteristic curves were employed to assess the discriminatory capacity of EZF expression (Supplementary Material 12). These findings support the suitability of the nomogram as a scoring tool for prognostic prediction in LAC patients.

## Discussion

Lung adenocarcinoma (LAC) is the most common subtype of lung cancer, yet the absence of reliable molecular markers continues to hinder precise prognostic prediction. The identification of novel biomarkers may improve risk stratification and guide individualized management.

The role of EZF in cancer biology is controversial. Some studies report that it is a tumor suppressor, but the results of the other studies support it is an oncogene. In colorectal and hepatocellular carcinoma, EZF suppresses tumor progression via modulating pathways such as RAB26 and JNK [26–28]. In contrast, EZF promotes stemness and metastasis in osteosarcoma and breast cancer [29, 30]. In non-small cell lung cancer, EZF has been reported to inhibit tumor proliferation via the JAK/STAT3 pathway [27]. However, these effects remain debated in the context of LAC, and its precise function warrants further exploration.

Playing a crucial role in maintaining lung homeostasis, EZF emerges as a significant contributor to cellular proliferation and differentiation [31, 32]. In this study, we observed higher levels of EZF expression in normal lung tissue compared to LAC, as evidenced by both the TCGA database and our collected samples, which is consistent with the characteristics of tumor suppressor and the reports of some previous studies [33]. However, paradoxically, increased EZF expression was also associated with advanced pathological stages (e.g., T3, T4) and poorer clinical outcomes. Kaplan–Meier survival analysis indicated that patients with higher EZF expression had significantly shorter OS, DSS, and PFI. Furthermore, both univariate and multivariate Cox analyses supported the prognostic value of EZF, suggesting its potential as an independent biomarker in LAC.

DNA methylation functions as an epigenetic mechanism that modulates the expression of functional genes [34]. The methylation status of the EZF promoter exerts diverse effects on developmental processes in both cancerous and healthy tissues. Our study aims to determine the extent of DNA methylation in EZF and investigate its correlation with clinical outcomes in LAC patients. Our findings revealed that most of the DNA sequences of EZF were less methylated in LAC. Importantly, we observed that increased levels of hypomethylation were associated with an unfavorable prognosis, particularly concerning EZF. These data suggest that the role of EZF in LAC progression is dynamically changing.

Beyond intrinsic tumor cell behaviors, increasing evidence points to EZF’s involvement in modulating the tumor immune microenvironment. We observed a strong correlation between EZF expression and immune infiltrates, particularly macrophages, mast cells, and eosinophils. Prior single-cell analyses have demonstrated that EGFR-mutant LACs are typically characterized by an immunosuppressive landscape [35]. Mechanistically, EZF has been implicated in promoting M2 macrophage polarization via STAT6 activation while suppressing pro-inflammatory M1 polarization through NF-κB inhibition [36, 37]. This dual role facilitates the establishment of an immunosuppressive tumor microenvironment that supports tumor progression and may contribute to resistance against immunotherapy.

Moreover, the regulation of EZF expression is highly context-dependent and modulated by multiple layers, including non-coding RNAs such as miR-34a-5p, which can suppress EZF to reverse M2 polarization and enhance antitumor immune responses [38]. Additional complexity arises from the subcellular localization of EZF, as nuclear and cytoplasmic distributions differentially impact its function and clinical outcomes in LAC [39]. Furthermore, EZF stability and activity are finely regulated by post-translational modifications, ubiquitination by enzymes such as USP10 [40], and interactions with oncogenic pathways [38], indicating that its role in the tumor immune microenvironment is intricately controlled at multiple levels. These findings underscore EZF’s multifaceted role as a molecular integrator of tumor-intrinsic and immune-mediated signals.

From a clinical perspective, incorporating EZF expression into prognostic models offers added value beyond conventional TNM staging. The proposed nomogram combines EZF expression with key clinical features such as pT stage, pN stage, and residual tumor status, allowing clinicians to better stratify patients into high- and low-risk categories. For instance, patients with intermediate-stage disease but high EZF expression might be reclassified into a higher-risk group, which could prompt more aggressive management strategies, such as closer surveillance or the use of adjuvant therapies. This personalized approach enhances the clinician’s ability to predict patient outcomes more accurately and could complement existing staging systems in guiding therapeutic decisions.

Despite the strengths of our integrative bioinformatic analysis and validation across multiple datasets, this study has limitations. Lack of prospective clinical specimens and experimental validation constrains mechanistic interpretations. Future work using in vitro and in vivo models is needed to dissect the functional roles of EZF in immune regulation and tumor progression. Moreover, absence of treatment-related clinical data limits our ability to assess EZF as a predictive biomarker for therapy response. Future research incorporating well-annotated patient cohorts will be essential to translate EZF into clinical practice.

## Conclusions

In conclusion, this study identifies EZF as a dual-function regulator in LAC, contributing to both tumor progression and immune modulation. Elevated EZF expression is associated with poorer clinical outcomes, including reduced overall and progression-free survival, and correlates with immune cell infiltration and epigenetic alterations within the tumor microenvironment. These findings highlight EZF as a promising prognostic biomarker and a potential therapeutic target. Further mechanistic and translational investigations are warranted to validate these insights and explore EZF-targeted strategies that may enhance precision oncology approaches in LAC. Importantly, integrating EZF into future clinical frameworks may facilitate personalized treatment decisions and accelerate its translation into routine oncological practice.

## Supplementary Information

Supplementary Figure S1. Protein expression of EZF was evaluated in both normal lung tissue and LAC, sourced from HPA database

Supplementary Figure S2. (A-D) Relationship between EZF expression and IPS score in four IPS groups: ips_ctla4_neg_pd1_neg, ips_ctla4_neg_pd1_pos, ips_ctla4_pos_pd1_neg, and ips_ctla4_pos_pd1_pos. (E) Relationship between EZF expression and TIDE score. TIDE, Tumor Immune Dysfunction and Exclusion; IPS, Immune Phenotype Scores

Supplementary Figure S3. External validation of the diagnostic and prognostic value of EZF in LAC. (A–B) ROC curves demonstrating the diagnostic performance of EZF in distinguishing LAC from normal lung tissues in two independent datasets, GSE10072 and GSE115002. (C–D) Kaplan–Meier survival analysis showing that high EZF expression is significantly associated with poorer overall survival in LAC patients from two additional external cohorts, GSE68465 and GSE68571

Supplementary Material 4

Supplementary Material 5

Supplementary Material 6

Supplementary Material 7

Supplementary Material 8

Supplementary Material 9

Supplementary Material 10

Supplementary Material 11

Supplementary Material 12

Supplementary Material 13

Supplementary Material 14

Supplementary Material 15

## Data Availability

Data of TCGA (https://portal.gdc.cancer.gov/) are available in public functional genomics data repositories. Other materials are available from the corresponding author via e-mail if the request is reasonable.
