# Supplementary figures and images for: Epithelial zinc finger protein in lung adenocarcinoma: prognostic biomarker with molecular and clinical implications
Source: Hereditas. 2025 Jun 18;162:106. doi: 10.1186/s41065-025-00476-7 (PMC12175355; doi:10.1186/s41065-025-00476-7)

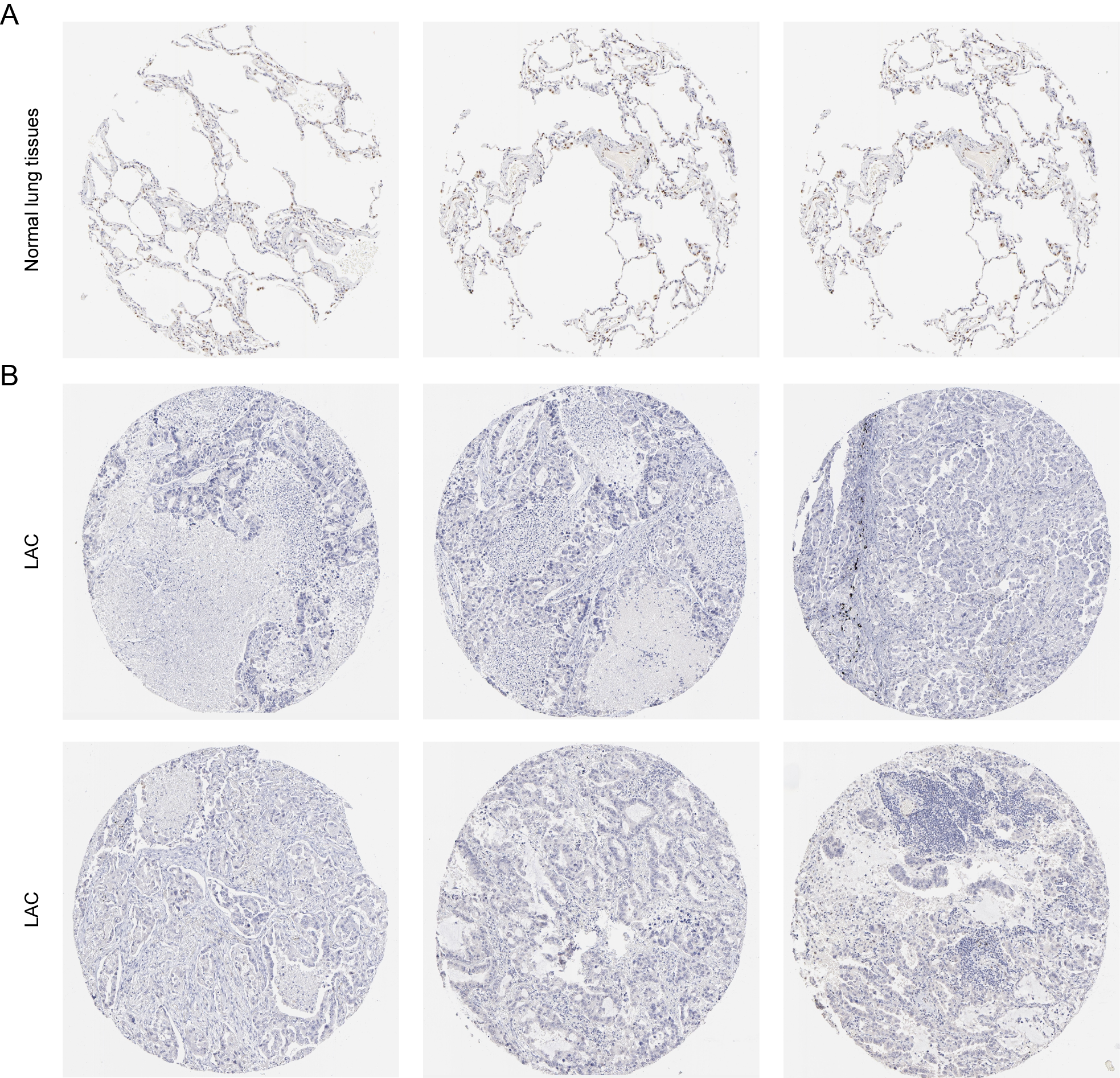

Supplement: Supplementary file 1 — Supplementary Figure S1. Protein expression of EZF was evaluated in both normal lung tissue and LAC, sourced from HPA database [file 41065_2025_476_MOESM1_ESM.tif]

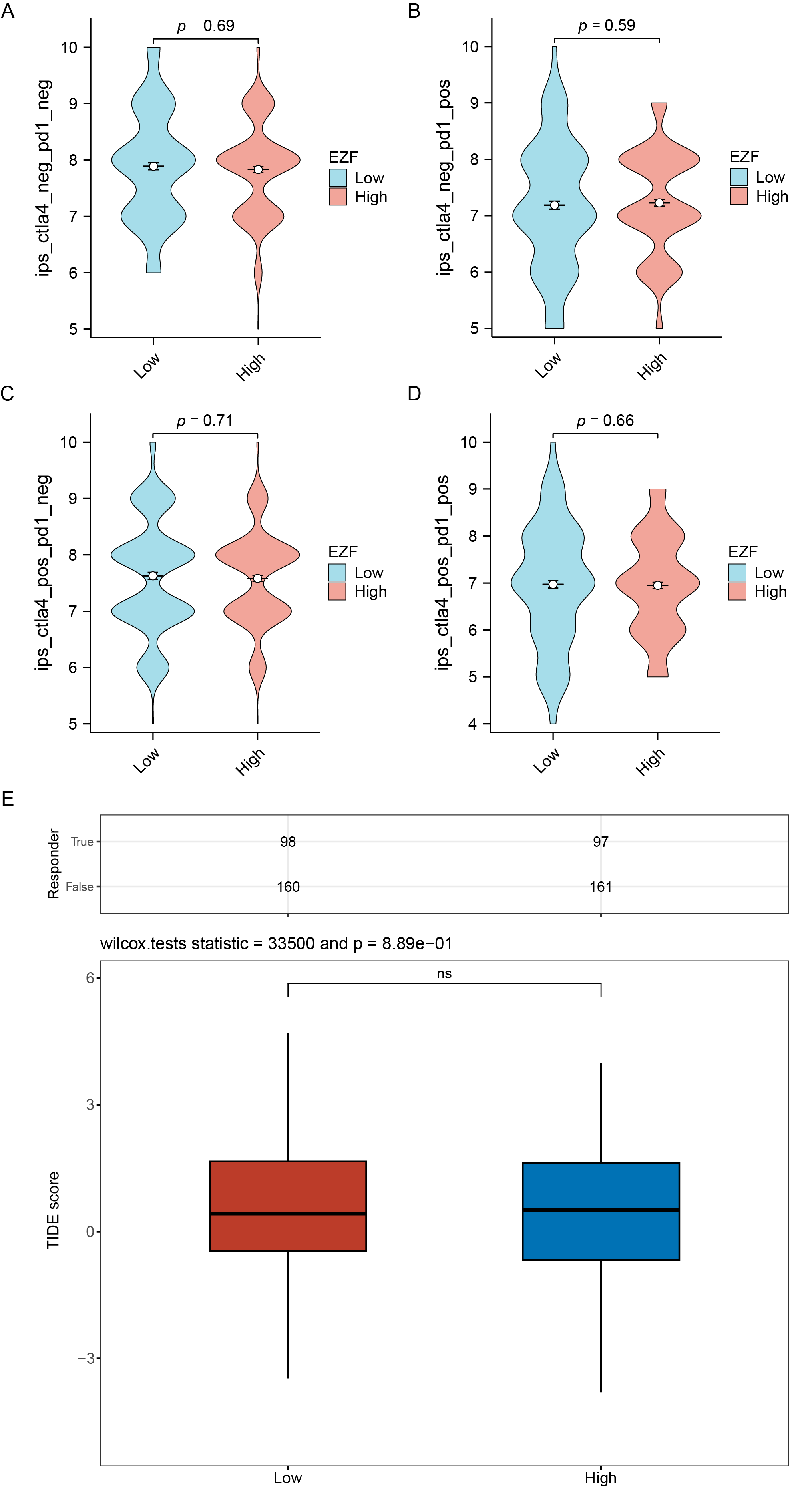

Supplement: Supplementary file 2 — Supplementary Figure S2. (A-D) Relationship between EZF expression and IPS score in four IPS groups: ips_ctla4_neg_pd1_neg, ips_ctla4_neg_pd1_pos, ips_ctla4_pos_pd1_neg, and ips_ctla4_pos_pd1_pos. (E) Relationship between EZF expression and TIDE score. TIDE, Tumor Immune Dysfunction and Exclusion; IPS, Immune Phenotype Scores [file 41065_2025_476_MOESM2_ESM.tif]

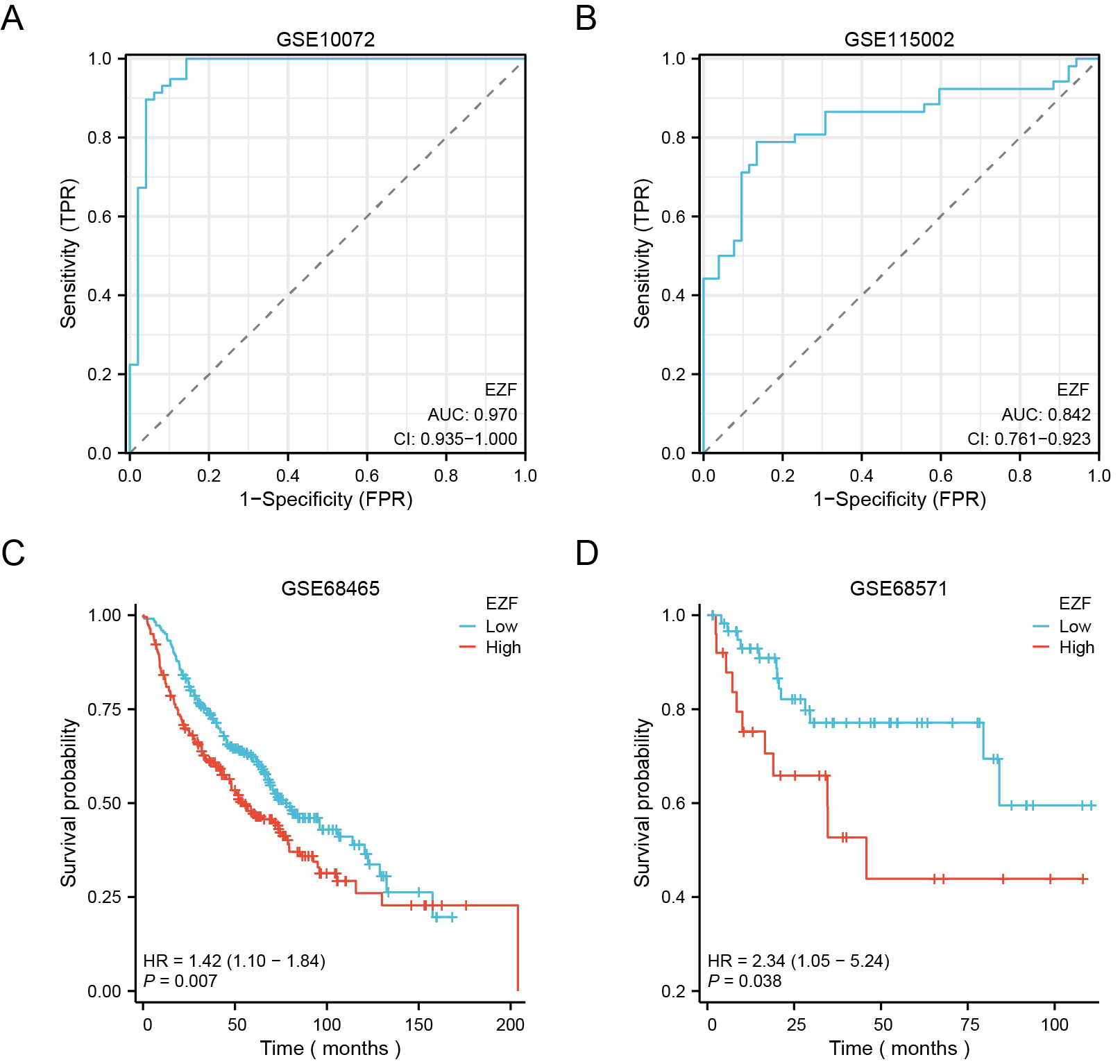

Supplement: Supplementary file 3 — Supplementary Figure S3. External validation of the diagnostic and prognostic value of EZF in LAC. (A–B) ROC curves demonstrating the diagnostic performance of EZF in distinguishing LAC from normal lung tissues in two independent datasets, GSE10072 and GSE115002. (C–D) Kaplan–Meier survival analysis showing that high EZF expression is significantly associated with poorer overall survival in LAC patients from two additional external cohorts, GSE68465 and GSE68571 [file 41065_2025_476_MOESM3_ESM.tif]
