# Supplementary material for: Epithelial zinc finger protein in lung adenocarcinoma: prognostic biomarker with molecular and clinical implications
Source: Hereditas. 2025 Jun 18;162:106. doi: 10.1186/s41065-025-00476-7 (PMC12175355; doi:10.1186/s41065-025-00476-7)
Supplement: Supplementary file 7 — Supplementary Material 7 [file 41065_2025_476_MOESM7_ESM.docx]

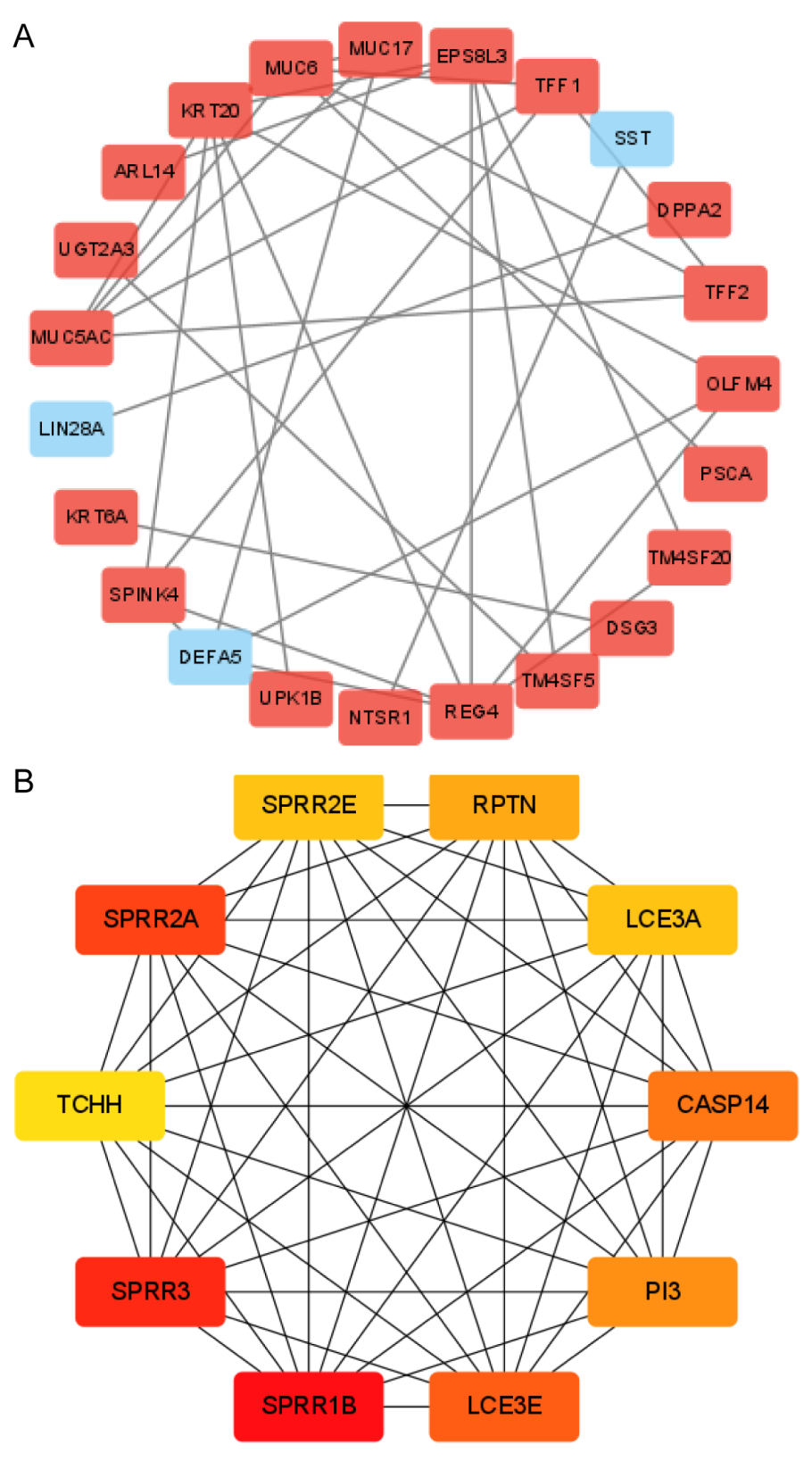


Supplementary Material 4. (A) Protein–protein interaction network of KLF4-related top 50 DEGs. Red and blue circles represent upregulated and downregulated DEGs, respectively. (B) Top 10 hub genes in KLF4 expression-associated DEGs. The red and yellow nodes represent the rank of hub genes from high to low according to the degree.
