# Supplementary material for: Epithelial zinc finger protein in lung adenocarcinoma: prognostic biomarker with molecular and clinical implications
Source: Hereditas. 2025 Jun 18;162:106. doi: 10.1186/s41065-025-00476-7 (PMC12175355; doi:10.1186/s41065-025-00476-7)
Supplement: Supplementary file 10 — Supplementary Material 10 [file 41065_2025_476_MOESM10_ESM.docx]

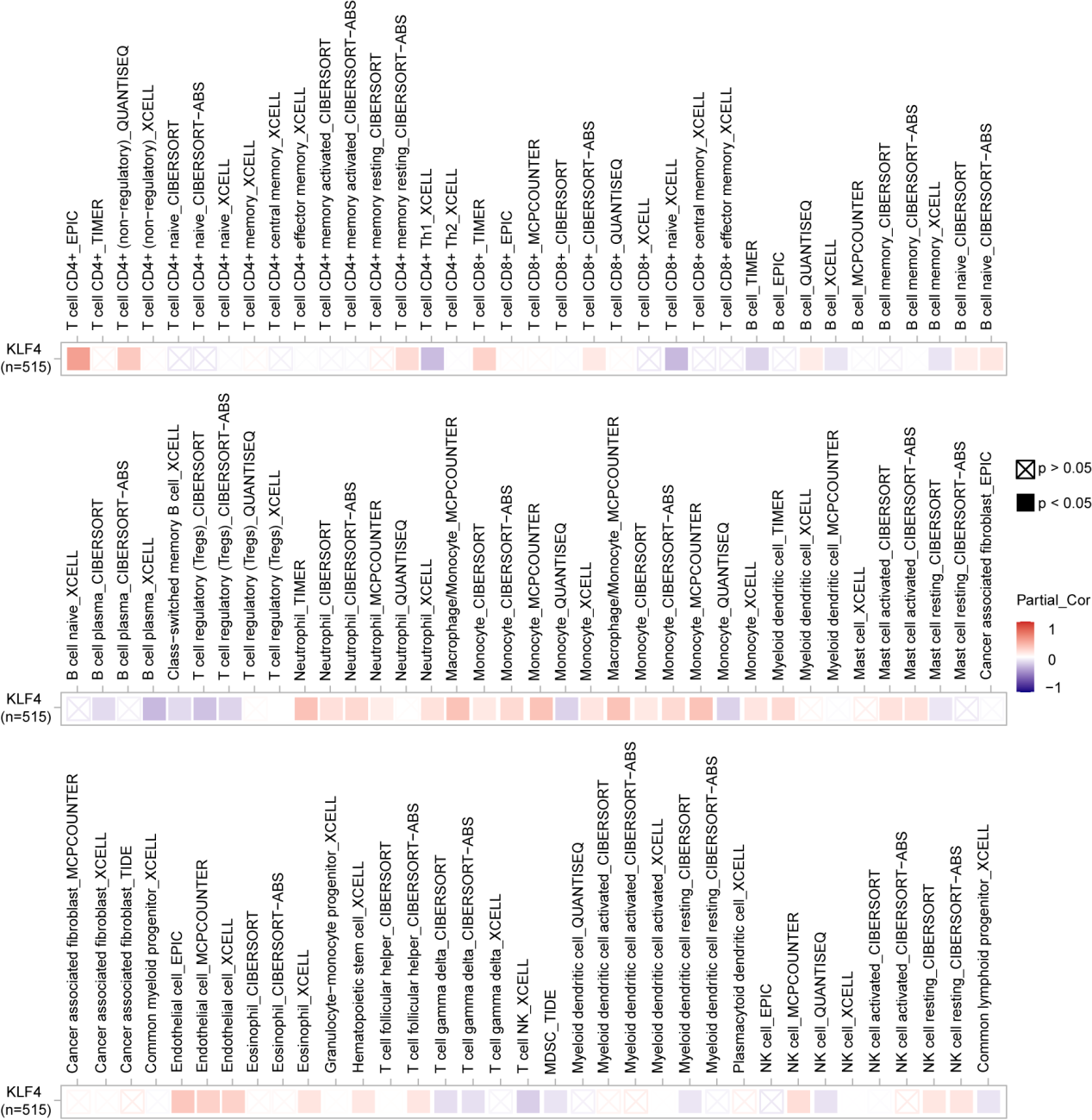


**Supplementary material 7.** The correlations of KLF4 expression and immune inﬁltration in LUAD from TIMER2.0 database.
