# Supplementary material for: Epithelial zinc finger protein in lung adenocarcinoma: prognostic biomarker with molecular and clinical implications
Source: Hereditas. 2025 Jun 18;162:106. doi: 10.1186/s41065-025-00476-7 (PMC12175355; doi:10.1186/s41065-025-00476-7)
Supplement: Supplementary file 11 — Supplementary Material 11 [file 41065_2025_476_MOESM11_ESM.docx]

**Supplementary Material 8. Univariate and multivariate analyses of overall survival in patients with LUAD.**

| Characteristics | Total (N) | Univariate analysis | | Multivariate analysis | |
| --- | --- | --- | --- | --- | --- |
|  |  | Hazard ratio  (95% CI) | *p* value | Hazard ratio  (95% CI) | *p* value |
| T stage | 501 |  |  |  |  |
| T1 | 168 | Reference |  |  |  |
| T2 | 269 | 1.454 (1.019-2.075) | 0.039 | 1.354 (0.824-2.224) | 0.232 |
| T3 | 45 | 2.954 (1.755-4.971) | <0.001 | 3.487 (1.555-7.822) | 0.002 |
| T4 | 19 | 3.176 (1.672-6.031) | <0.001 | 2.022 (0.774-5.277) | 0.151 |
| N stage | 492 |  |  |  |  |
| N0 | 325 | Reference |  |  |  |
| N1 | 94 | 2.387 (1.692-3.366) | <0.001 | 2.159 (1.035-4.503) | 0.04 |
| N2&N3 | 73 | 2.974 (2.037-4.343) | <0.001 | 2.162 (0.783-5.971) | 0.137 |
| M stage | 360 |  |  |  |  |
| M0 | 335 | Reference |  |  |  |
| M1 | 25 | 2.111 (1.232-3.616) | 0.007 | 1.251 (0.461-3.392) | 0.66 |
| Pathologic stage | 496 |  |  |  |  |
| Stage I | 270 | Reference |  |  |  |
| Stage II | 119 | 2.469 (1.716-3.552) | <0.001 | 0.809 (0.370-1.770) | 0.596 |
| Stage III | 81 | 3.567 (2.438-5.218) | <0.001 | 1.468 (0.479-4.496) | 0.501 |
| Stage IV | 26 | 3.813 (2.197-6.618) | <0.001 |  |  |
| Residual tumor | 352 |  |  |  |  |
| R0 | 336 | Reference |  |  |  |
| R1&R2 | 16 | 3.973 (2.217-7.120) | <0.001 | 2.253 (0.999-5.080) | 0.05 |
| Anatomic neoplasm subdivision | 490 |  |  |  |  |
| Left | 194 | Reference |  |  |  |
| Right | 296 | 1.024 (0.758-1.383) | 0.878 |  |  |
| Anatomic neoplasm subdivision2 | 182 |  |  |  |  |
| Central Lung | 62 | Reference |  |  |  |
| Peripheral Lung | 120 | 0.913 (0.570-1.463) | 0.706 |  |  |
| Gender | 504 |  |  |  |  |
| Female | 270 | Reference |  |  |  |
| Male | 234 | 1.060 (0.792-1.418) | 0.694 |  |  |
| Race | 446 |  |  |  |  |
| White | 387 | Reference |  |  |  |
| Asian&Black or African American | 59 | 0.703 (0.430-1.151) | 0.162 |  |  |
| Age | 494 |  |  |  |  |
| <=65 | 238 | Reference |  |  |  |
| >65 | 256 | 1.228 (0.915-1.649) | 0.171 |  |  |
| Smoker | 490 |  |  |  |  |
| No | 71 | Reference |  |  |  |
| Yes | 419 | 0.887 (0.587-1.339) | 0.568 |  |  |
| number_pack_years  _smoked | 345 |  |  |  |  |
| <40 | 169 | Reference |  |  |  |
| >=40 | 176 | 1.038 (0.723-1.490) | 0.840 |  |  |
| KLF4 | 504 |  |  |  |  |
| Low | 255 | Reference |  |  |  |
| High | 249 | 1.395 (1.042-1.868) | 0.025 | 1.867 (1.265-2.755) | 0.002 |

Abbreviations: KLF4, Kruppel-like factor 4; CI, confidence interval.
