# Supplementary material for: Epithelial zinc finger protein in lung adenocarcinoma: prognostic biomarker with molecular and clinical implications
Source: Hereditas. 2025 Jun 18;162:106. doi: 10.1186/s41065-025-00476-7 (PMC12175355; doi:10.1186/s41065-025-00476-7)
Supplement: Supplementary file 12 — Supplementary Material 12 [file 41065_2025_476_MOESM12_ESM.docx]

**Supplementary Material 9. Univariate and multivariate analyses of disease-specific survival in patients with LUAD.**

| **Characteristics** | **Total (N)** | **Univariate analysis** | | **Multivariate analysis** | |
| --- | --- | --- | --- | --- | --- |
|  |  | **Hazard ratio**  **(95% CI)** | ***p* value** | **Hazard ratio**  **(95% CI)** | ***p* value** |
| T stage | 466 |  |  |  |  |
| T1 | 161 | Reference |  |  |  |
| T2 | 249 | 1.585 (1.007-2.497) | 0.047 | 1.263 (0.666-2.396) | 0.474 |
| T3 | 41 | 2.864 (1.462-5.612) | 0.002 | 2.572 (0.815-8.117) | 0.107 |
| T4 | 15 | 2.628 (1.007-6.858) | 0.048 | 1.417 (0.324-6.203) | 0.643 |
| N stage | 457 |  |  |  |  |
| N0 | 309 | Reference |  |  |  |
| N1 | 83 | 2.845 (1.855-4.365) | <0.001 | 1.983 (0.759-5.180) | 0.162 |
| N2&N3 | 65 | 2.720 (1.660-4.456) | <0.001 | 1.886 (0.377-9.422) | 0.440 |
| M stage | 327 |  |  |  |  |
| M0 | 306 | Reference |  |  |  |
| M1 | 21 | 2.480 (1.278-4.811) | 0.007 | 1.723 (0.547-5.432) | 0.353 |
| Pathologic stage | 461 |  |  |  |  |
| Stage I | 257 | Reference |  |  |  |
| Stage II | 110 | 3.252 (2.049-5.163) | <0.001 | 1.080 (0.384-3.036) | 0.884 |
| Stage III | 72 | 3.523 (2.118-5.860) | <0.001 | 1.590 (0.281-8.980) | 0.600 |
| Stage IV | 22 | 4.907 (2.485-9.691) | <0.001 |  |  |
| Residual tumor | 320 |  |  |  |  |
| R0 | 307 | Reference |  |  |  |
| R1&R2 | 13 | 5.020 (2.479-10.167) | <0.001 | 3.318 (1.115-9.871) | 0.031 |
| Anatomic neoplasm subdivision | 456 |  |  |  |  |
| Left | 179 | Reference |  |  |  |
| Right | 277 | 1.109 (0.749-1.641) | 0.606 |  |  |
| Anatomic neoplasm subdivision2 | 167 |  |  |  |  |
| Central Lung | 57 | Reference |  |  |  |
| Peripheral Lung | 110 | 1.207 (0.630-2.312) | 0.570 |  |  |
| Gender | 469 |  |  |  |  |
| Female | 252 | Reference |  |  |  |
| Male | 217 | 0.956 (0.658-1.390) | 0.815 |  |  |
| Race | 423 |  |  |  |  |
| White | 365 | Reference |  |  |  |
| Asian&Black or African American | 58 | 0.918 (0.531-1.587) | 0.759 |  |  |
| Age | 459 |  |  |  |  |
| <=65 | 226 | Reference |  |  |  |
| >65 | 233 | 1.039 (0.713-1.513) | 0.842 |  |  |
| Smoker | 455 |  |  |  |  |
| No | 68 | Reference |  |  |  |
| Yes | 387 | 1.013 (0.585-1.755) | 0.962 |  |  |
| number_pack_years_  smoked | 318 |  |  |  |  |
| <40 | 158 | Reference |  |  |  |
| >=40 | 160 | 0.904 (0.569-1.437) | 0.670 |  |  |
| KLF4 | 469 |  |  |  |  |
| Low | 239 | Reference |  |  |  |
| High | 230 | 1.561 (1.072-2.274) | 0.020 | 1.845 (1.086-3.133) | 0.024 |

Abbreviations: KLF4, Kruppel-like factor 4; CI, confidence interval.
