# Supplementary material for: Epithelial zinc finger protein in lung adenocarcinoma: prognostic biomarker with molecular and clinical implications
Source: Hereditas. 2025 Jun 18;162:106. doi: 10.1186/s41065-025-00476-7 (PMC12175355; doi:10.1186/s41065-025-00476-7)
Supplement: Supplementary file 13 — Supplementary Material 13 [file 41065_2025_476_MOESM13_ESM.docx]

**Supplementary material 10. Univariate and multivariate analyses of progress-free interval in patients with LUAD.**

| **Characteristics** | **Total (N)** | **Univariate analysis** | | **Multivariate analysis** | |
| --- | --- | --- | --- | --- | --- |
|  |  | **Hazard ratio**  **(95% CI)** | ***p* value** | **Hazard ratio**  **(95% CI)** | ***p* value** |
| T stage | 501 |  |  |  |  |
| T1 | 168 | Reference |  |  |  |
| T2 | 269 | 1.686 (1.210-2.349) | **0.002** | 1.404 (0.879-2.244) | 0.156 |
| T3 | 45 | 3.548 (2.195-5.735) | **<0.001** | 2.584 (1.106-6.038) | **0.028** |
| T4 | 19 | 1.129 (0.449-2.837) | 0.797 | 0.659 (0.182-2.387) | 0.526 |
| N stage | 492 |  |  |  |  |
| N0 | 325 | Reference |  |  |  |
| N1 | 94 | 1.700 (1.226-2.356) | **0.001** | 0.962 (0.476-1.947) | 0.915 |
| N2&N3 | 73 | 1.619 (1.099-2.383) | **0.015** | 0.597 (0.160-2.234) | 0.444 |
| M stage | 360 |  |  |  |  |
| M0 | 335 | Reference |  |  |  |
| M1 | 25 | 1.620 (0.913-2.876) | 0.099 | 1.477 (0.590-3.701) | 0.405 |
| Pathologic stage | 496 |  |  |  |  |
| Stage I | 270 | Reference |  |  |  |
| Stage II | 119 | 2.222 (1.609-3.067) | **<0.001** | 1.507 (0.728-3.123) | 0.269 |
| Stage III | 81 | 2.032 (1.382-2.988) | **<0.001** | 2.544 (0.645-10.026) | 0.182 |
| Stage IV | 26 | 2.337 (1.324-4.125) | **0.003** |  |  |
| Residual tumor | 352 |  |  |  |  |
| R0 | 336 | Reference |  |  |  |
| R1&R2 | 16 | 3.303 (1.771-6.160) | **<0.001** | 3.196 (1.420-7.196) | **0.005** |
| Anatomic neoplasm subdivision | 490 |  |  |  |  |
| Left | 194 | Reference |  |  |  |
| Right | 296 | 1.067 (0.801-1.421) | 0.658 |  |  |
| Anatomic neoplasm subdivision2 | 182 |  |  |  |  |
| Central Lung | 62 | Reference |  |  |  |
| Peripheral Lung | 120 | 1.073 (0.673-1.713) | 0.766 |  |  |
| Gender | 504 |  |  |  |  |
| Female | 270 | Reference |  |  |  |
| Male | 234 | 1.072 (0.815-1.411) | 0.617 |  |  |
| Race | 446 |  |  |  |  |
| White | 387 | Reference |  |  |  |
| Asian&Black or African American | 59 | 0.915 (0.604-1.385) | 0.673 |  |  |
| Age | 494 |  |  |  |  |
| <=65 | 238 | Reference |  |  |  |
| >65 | 256 | 1.106 (0.837-1.460) | 0.479 |  |  |
| Smoker | 490 |  |  |  |  |
| No | 71 | Reference |  |  |  |
| Yes | 419 | 0.946 (0.637-1.404) | 0.782 |  |  |
| number_pack_years_  smoked | 345 |  |  |  |  |
| <40 | 169 | Reference |  |  |  |
| >=40 | 176 | 1.011 (0.723-1.414) | 0.950 |  |  |
| KLF4 | 504 |  |  |  |  |
| Low | 255 | Reference |  |  |  |
| High | 249 | 1.319 (1.002-1.736) | **0.048** | 1.547 (1.057-2.265) | **0.025** |

Abbreviations: KLF4, Kruppel-like factor 4; CI, confidence interval.
