# Supplementary material for: Epithelial zinc finger protein in lung adenocarcinoma: prognostic biomarker with molecular and clinical implications
Source: Hereditas. 2025 Jun 18;162:106. doi: 10.1186/s41065-025-00476-7 (PMC12175355; doi:10.1186/s41065-025-00476-7)
Supplement: Supplementary file 14 — Supplementary Material 14 [file 41065_2025_476_MOESM14_ESM.docx]

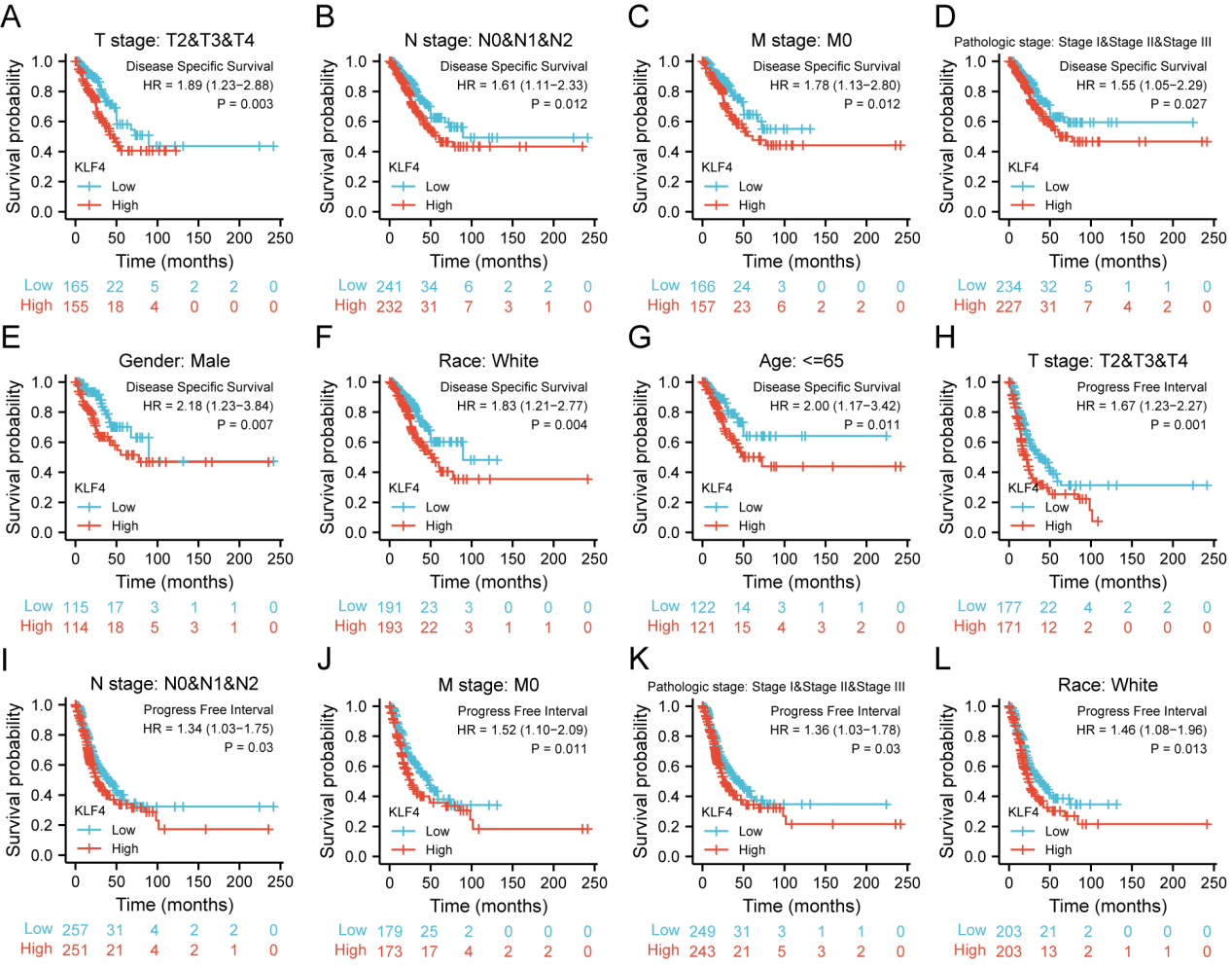


Supplementary Material 11. Prognostic values of KLF4 expression in patients with LUAD evaluated by the Kaplan-Meier method in different subgroups. (A–G) DSS survival curves of T2 & T3 &T4, N0 &N1 & N2, M0, stage I &II & III, Male, White, age <=65 years. (H–L) PFI survival curves of T2 & T3 &T4, N0 &N1 & N2, M0, stage I &II & III, White.

DSS, disease-speciﬁc survival; PFI, Progress free interval.
