# Supplementary material for: Epithelial zinc finger protein in lung adenocarcinoma: prognostic biomarker with molecular and clinical implications
Source: Hereditas. 2025 Jun 18;162:106. doi: 10.1186/s41065-025-00476-7 (PMC12175355; doi:10.1186/s41065-025-00476-7)
Supplement: Supplementary file 15 — Supplementary Material 15 [file 41065_2025_476_MOESM15_ESM.docx]

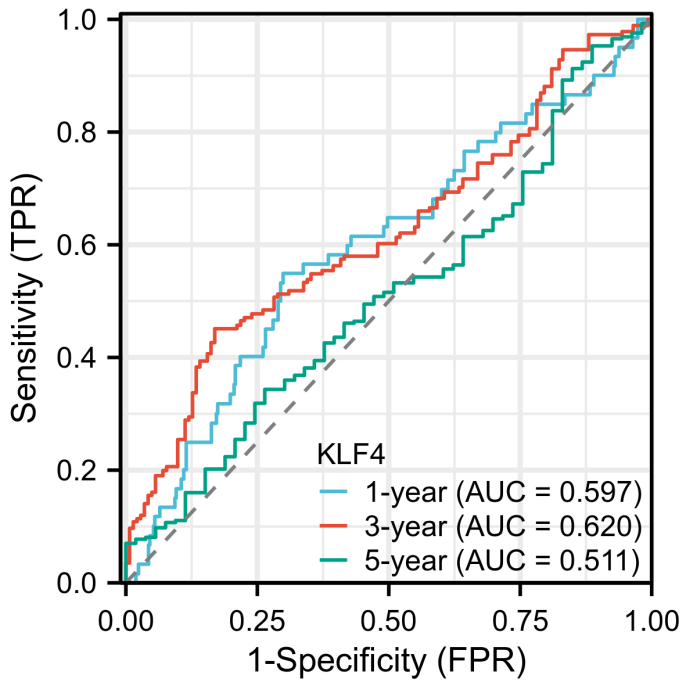


**Supplementary Material 12.** Time-dependent ROC curves at different time points Time-dependent ROC curves based on *KLF4* expression for one-, three-, and five-year OS probability. ROC, receiver-operating characteristic; AUC, area under the ROC curve; OS, overall survival.
